# Assembly of high-nuclearity **Sn_26_**, **Sn_34_**-oxo clusters: solvent strategies and inorganic Sn incorporation†
†Electronic supplementary information (ESI) available: Experimental details, images of the structures, IR and UV-vis spectra, PXRD patterns, TG curves and the calibration curve for formate and faradaic efficiencies of H_2_. CCDC 1563679, 1563680, 1563685 and 1895007–1895010. For ESI and crystallographic data in CIF or other electronic format see DOI: 10.1039/c9sc02503k


**DOI:** 10.1039/c9sc02503k

**Published:** 2019-08-12

**Authors:** Yu Zhu, Lei Zhang, Jian Zhang

**Affiliations:** a State Key Laboratory of Structural Chemistry , Fujian Institute of Research on the Structure of Matter , Chinese Academy of Sciences , Fuzhou , Fujian 350002 , P. R. China . Email: LZhang@fjirsm.ac.cn

## Abstract

A series of unprecedented high-nuclearity tin-oxo nanoclusters (up to **Sn_34_**) with structural diversity have been obtained. The characteristics of the applied solvents had great influence on the assembly of these Sn–O clusters. Pure alcohol environments only gave rise to small clusters of **Sn_6_**, whilst the introduction of water significantly increased the nuclearity to **Sn_26_**, which greatly exceeds those of the known tin-oxo clusters (≤14); the use of aprotic CH_3_CN finally produced the largest **Sn_34_** to date. Apart from the nuclearity breakthrough, the obtained tin-oxo clusters also present new structural types that are not found in previous reports, including a layered nanorod-like structure of **Sn_26_** and the cage-dimer structure of **Sn_34_**. The layered **Sn_26_** clusters represent good molecular models for SnO_2_ materials. Moreover, an electrode derived from **TOC-17** with a {**Sn_26_**} core shows better electrocatalytic CO_2_ reduction activity than that from **TOC-18** with **Sn_34_**. This work not only provides an efficient methodology for the rational assembly of high-nuclearity Sn–O clusters, but also extends their potential applications in energy conversion.

## Abstract 2

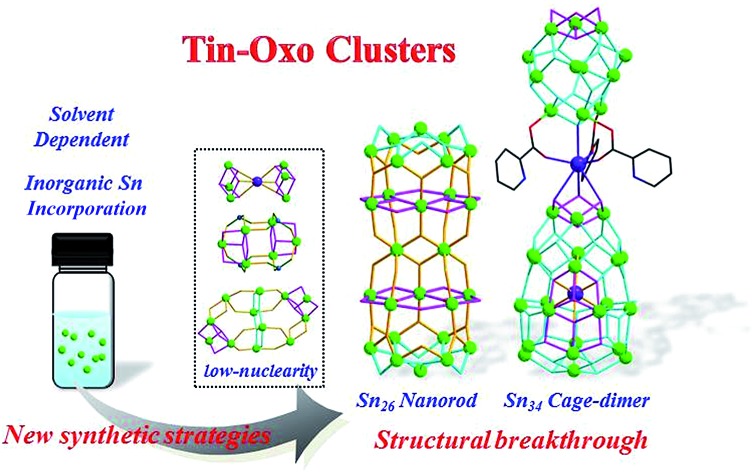
Unprecedented tin-oxo clusters with record high-nuclearities and electrocatalytic CO_2_ reduction applications have been prepared *via* solvent dependent assembly strategies.

## Introduction

Tin oxide (SnO_2_) has attracted increasing research attention due to its application in a variety of areas, including gas sensors,^1^ catalysis,^2^,^3^ lithium batteries,^4^,^5^ solar cells^6^,^7^ and transparent electrodes.^8^ Some important factors, such as the size, composition, and structure, greatly influence the electronic and physicochemical properties of SnO_2_.^9^–^11^ Thus it is crucial to understand the binding mode and atomic connectivity of tin oxide materials at the molecular level, which will be beneficial for exploring the structure–property relationship and further achieving precise tuning of the physicochemical properties.

As molecular models of tin oxide materials, crystalline tin-oxo clusters (TOCs) can provide precise atomic structural information by X-ray diffraction analysis. Accordingly, they are efficient molecular tools for building bridges between theoretical modeling, crystallography, and physical applications. Many research groups have made great efforts to explore the synthesis and structures of TOCs.^12^–^22^ One common way is to use organotin as precursors to react with carboxylate ligands in organic solvents, such as benzene, toluene, and alcohols.^23^–^27^ To date, the representative structural types of TOCs are characterized by cubic [*n*-BuSn(O)O_2_P(*t*-Bu)_2_]_4_,^28^ hexameric drum {R′SnO(O_2_CR)}_6_,^29^ ladder [(R′Sn(O)O_2_CR)_2_R′Sn(O_2_CR)_3_]_2_,^30^ and Keggin-type alkyltins.^31^–^33^ However, despite these advances, the structural diversity of tin-oxo clusters is still less developed compared to transition-metal oxo clusters. And the TOC nuclearity also remains quite low, with the highest one being **Sn_14_**.^34^ Therefore, it is an attractive but challenging goal to synthesize TOCs with larger core nuclearity and more structural diversity. Moreover, although most of the driving forces of the research on TOCs originate from understanding the physical attributes of tin oxide, the studies on their applications (*e.g.* in catalysis) still remain very rare. Thus it is rather urgent to explore the physical application of TOCs to acquire the important Sn–O connectivity–activity relationship.

The assembly of tin-oxo clusters highly depends on the reactivity of organotin precursors, which is further influenced by organic ligands, reaction temperature, solvent and so on. Therefore, to prepare a diverse range of high-nuclearity TOCs, it is necessary to develop new synthetic approaches. During our recent research in titanium-oxo clusters,^35^–^37^ high-temperature assembly reactions were widely used for the preparation of high nuclearity Ti–O clusters. Herein, we introduce this method into the field of tin-oxo clusters, and make the following indispensable modifications: (1) mixed alcohol–water solvents were applied to influence the aggregation rate of Sn atoms and the nucleation rate of tin-oxo clusters; (2) the aprotic solvent CH_3_CN was then used in some cases instead of the general protic solvent to affect the configuration of basic building blocks, as well as their way of connecting; (3) finally, inorganic Sn atoms without any alkyl group or phenyl group were introduced into the reaction system to supply more bridging Sn atoms to promote the formation of high nuclearity TOCs. With these synthetic strategies, a series of unprecedented high-nuclearity TOCs have been successfully obtained whose atomic structures were characterized by single crystal X-ray diffraction analysis (Table 1). They possess a much higher number of Sn atoms (26, 34) than the known TOCs (≤14). Meanwhile, they also present new structural types different from previous TOCs, including the rod-shaped **Sn_26_** and cage-dimer **Sn_34_**. Furthermore, the application of **Sn_26_** or **Sn_34_** derived electrode in electrocatalytic CO_2_ reduction was investigated for the first time. Nuclear magnetic resonance (NMR) spectroscopy analysis indicated that formate was obtained as the only liquid reduction product. And the corresponding formate faradaic efficiency (FE) was found to be cluster dependent, with the highest value of 41.90% on the **Sn_26_** derived electrode.

**Table 1:** Summary of the composition and reaction conditions of the obtained TOCs^*a*^

Compound	Formula	Sn source	Solvent	Nuclearity
**TOC-12**	NaH[(*n*-BuSn)_3_(PDC)_3_(OCH_3_)(OH)_3_]_2_·6CH_3_OH	Butyltin hydroxide oxide	Methanol–isopropanol	**Sn_6_**
**TOC-13**	(*n*-BuSn)_6_O_2_(PP)_4_(OCH_3_)_6_	Butyltin hydroxide oxide	Methanol–isopropanol	**Sn_6_**
**TOC-14**	[(*n*-BuSn)_12_(OH)_18_O_4_(BPA)_2_(H_2_BPA)_4_]·6H_2_O	Butyltin hydroxide oxide	Methanol–water	**Sn_12_**
**TOC-15**	[(*n*-BuSn)_22_Sn_4_(OH)_26_O_22_(TZAC)_6_]·4Cl·2(TZAC)·2(CH_3_OH)·8H_2_O	Butyltin hydroxide oxide	Methanol–isopropanol–water	**Sn_26_**
**TOC-16**	[(*n*-BuSn)_22_Sn_4_(OH)_26_O_22_(IANO)_6_]·6(IANO)·6(CH_3_OH)·4H_2_O	Butyltin hydroxide oxide	Methanol–isopropanol–water	**Sn_26_**
**TOC-17**	[(*n*-BuSn)_22_Sn_4_(OH)_26_O_22_(IANO)_6_][(*n*-BuSn)_2_(OH)_2_(IANO)Cl_4_]_2_·4Cl·6CH_3_OH·10H_2_O	Butyltin hydroxide oxide and SnCl_4_	Methanol–isopropanol–water	**Sn_26_**
**TOC-18**	[(*n*-BuSn)_34_Na_2_(OH)_14_O_40_(PA)_8_]·2(PA)·8H_2_O	Butyltin hydroxide oxide	Acetonitrile	**Sn_34_**

^*a*^ *n*-Bu = butyl group; PDC = 2,6-pyridinedicarboxylate; PP = phenylphosphonate; H_3_BPA = 2,2-bis(hydroxymethyl)propionic acid; HTZAC = 1*H*-tetrazole-1-acetic acid; HIANO = isonicotinic acid-*N*-oxide; HPA = 2-picolinic acid.

## Results and discussion

**Fig. 1:**
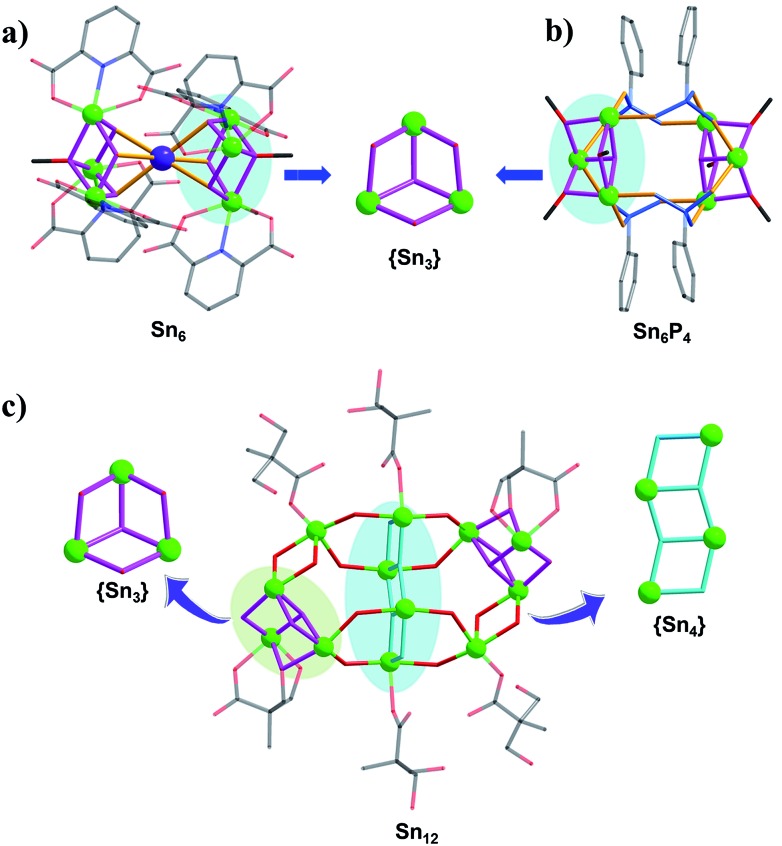
The ball-and-stick representations of **TOC-12** (a), **TOC-13** (b) and **TOC-14** (c). Atom color code: green Sn; purple Na; red O; black C; dark blue N; light blue P.

In the initial stage of our research, mixed methanol–isopropanol was used as the solvent for reactions between butyltin hydroxide oxide and 2,6-pyridinedicarboxylic acid/NaOH or phenylphosphonic acid, resulting in the formation of two **Sn_6_** clusters (**TOC-12** and **TOC-13**). Structural analysis indicated that they were both composed of two O-capped {Sn_3_O_4_} units (Fig. 1a and b; Table 1). Different from the back-to-back linking mode of two O-capped {Sn_3_O_4_} units *via* PP ligands in **TOC-13**, the O-capped {Sn_3_O_4_} units in **TOC-12** are linked by a Na atom in a face-to-face fashion to form a sandwich-like {Sn_3_NaSn_3_} architecture. It is notable that methanol plays a bridging role in the O-capped {Sn_3_O_4_} units of **TOC-12** and **TOC-13**, implying that the strong coordination ability and small steric hindrance of methanol may be in favour of the formation of the O-capped {Sn_3_O_4_} unit. In order to obtain different building units and change the aggregation/nucleation rate of tin atoms, water which is crucial for the growth of oxo clusters was directly introduced into the reaction system. Consequently, a new **Sn_12_** TOC was prepared namely [(*n*-BuSn)_12_(OH)_18_O_4_(BPA)_2_(H_2_BPA)_4_]·6H_2_O (**TOC-14**) (H_3_BPA = 2,2-bis(hydroxymethyl)propionic acid). As shown in Fig. 1c, **TOC-14** is constructed from two different building blocks, the O-capped {Sn_3_O_4_} and ladder {Sn_4_O_4_} units, indicating that the introduction of water is an effective strategy to prepare different structural types of TOCs with higher nuclearity.

**Fig. 2:**
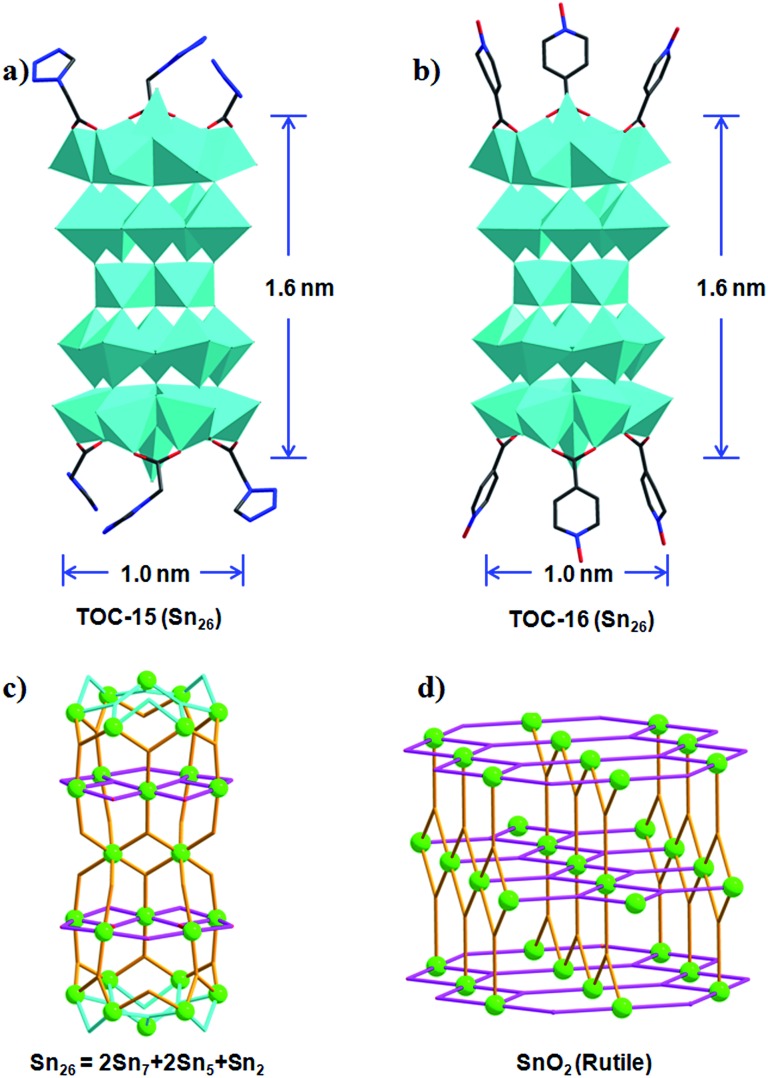
Polyhedral representations of the **Sn_26_** cluster in **TOC-15** (a) and **TOC-16** (b). The ball-and-stick representations of **Sn_26_** (c) and SnO_2_ (rutile) (d). Polyhedral color code: blue SnO_5_C/SnO_3_C/SnO_6_. Atom color code: green Sn.

By further optimizing the reaction conditions, especially the amount of used water, **TOC-15** and **TOC-16** with nuclearities of **Sn_26_** were successfully obtained in a mixed solvent system of methanol–isopropanol–water (Table 1, Fig. 2). To the best of our knowledge, the numbers of Sn atoms (26) in these TOCs far exceed the value (≤14) in reported tin-oxo clusters to date. The core size of these nanoclusters is ∼1.6 × 1.0 nm. As shown in Fig. 2, different from the usual cage structures of reported TOCs, **TOC-15** presents a rod-shaped cluster core that can be considered to be made up of five tin-oxo layers, including two planar {**Sn_5_**} units, two capped {**Sn_7_**} moieties and one {**Sn_2_**} dimer. The {**Sn_7_**} and {**Sn_5_**} units are linked together by five μ_3_-O atoms to form a {**Sn_12_**} moiety. And two symmetry-related {**Sn_12_**} moieties are further held together through the central {**Sn_2_**} dimer to give the **Sn_26_** core of **TOC-15**, which is further protected by six TZAC ligands. Such a layered structure type is found for the first time in the area of TOCs, and is reminiscent of the tin-oxo layers in SnO_2_ (Fig. 2c and d). By changing the functional ligands, other **Sn_26_** clusters of **TOC-16** were obtained with the six labile coordination sites decorated with IANO ligands (Fig. S6†).

Although **TOC-15** and **TOC-16** present interesting high-nuclearity layered structures, their yields are unfortunately quite low (2–4%), which greatly limits further applications. To obtain some mechanistic information of such synthetic shortages, we analyzed their structures in more detail. It is interesting to find that **TOC-15** and **TOC-16** contain pure inorganic SnO_6_ nodes without butyl groups. Such completely O-coordinated Sn atoms should be derived from the Sn–C bond cleavage of the applied butyltin hydroxide oxide. Considering the difficulty of Sn–C bond cleavage under the applied low-temperature conditions, it will benefit the assembly of such Sn–O cores if isolated Sn ions could be incorporated into the reactions. For this aim, the inorganic SnCl_4_ precursor was further introduced into the synthetic reaction of **TOC-16**. As expected, **TOC-17** was successfully isolated in a much higher yield (∼60%). As presented in Fig. S8,† **TOC-17** is composed of the same **Sn_26_** moiety in **TOC-16** and two additional {**Sn_2_**} dimers which are made up of two Sn atoms bridged by two oxygen atoms and one IANO ligand. The successful preparation of **TOC-17** demonstrates that the strategy of introducing additional inorganic Sn atoms is indeed helpful for the formation of high-nuclearity TOCs.

**Fig. 3:**
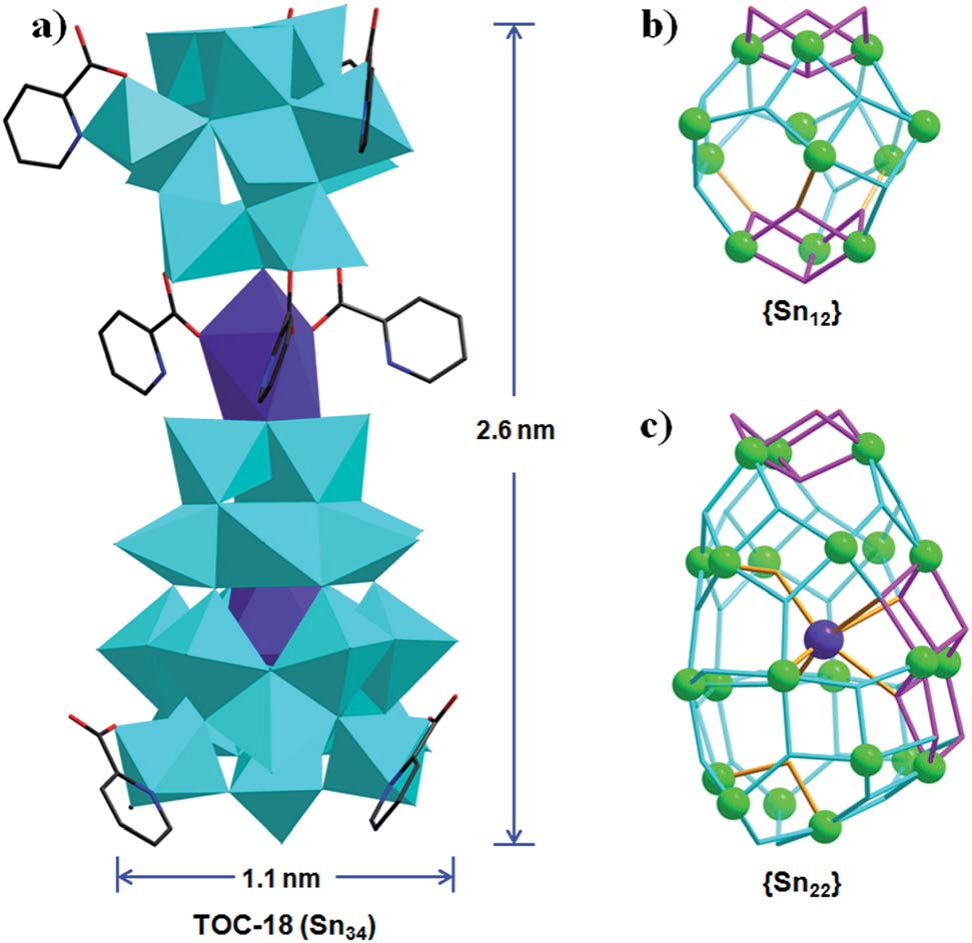
(a) The polyhedral representation of **TOC-18**. (b) and (c) The ball-and-stick representations of {**Sn_12_**} and {**Sn_22_**} moieties in **TOC-18**. Polyhedral color code: blue SnO_5_C/SnO_4_C/SnO_4_NC; purple NaO_7_/NaO_6_. Atom color code: green Sn; purple Na; red O; black C; dark blue N.

Based on the above results, we can clearly see that the assembly of Sn–O clusters is greatly influenced by the applied solvent conditions. Pure alcohol environments gave rise to small clusters of **Sn_6_**, and the introduction of water could significantly increase the nuclearities to **Sn_26_** (Table 1).^38^ However, as a whole, the above used solvents are all protic ones. If an aprotic solvent, *e.g.* CH_3_CN, could be applied, the different solvent environment may change the configuration of basic Sn–O building units, as well as their way of connecting. Following this consideration, the reaction of butyltin hydroxide oxide with 2-picolinic acid and NaOH was carried out in pure CH_3_CN. As a consequence, **TOC-18** with a nuclearity of **Sn_34_** and a core size of ∼2.6 × 1.1 nm was successfully synthesized, which is the largest Sn–O cluster reported to date. As exhibited in Fig. 3a, **TOC-18** is made up of two unprecedented {**Sn_12_**} and {**Sn_22_**} cages linked together by a Na atom and PA ligands. Compared with the typical football cage {(RSn)_12_O_14_(OH)_6_}^2–^, the {**Sn_12_**} in **TOC-18** displays an asymmetric cage-like structure (Fig. 3b). The {**Sn_22_**} cage captures a central Na heteroatom *via* six oxygen atoms (Fig. 3c). From an architectural point of view, the {**Sn_22_**} cage can be considered to consist of four subunits with different numbers of Sn atoms, including a top **Sn_3_** moiety, two middle **Sn_6_** circles and a bottom **Sn_7_** base.

**Fig. 4:**
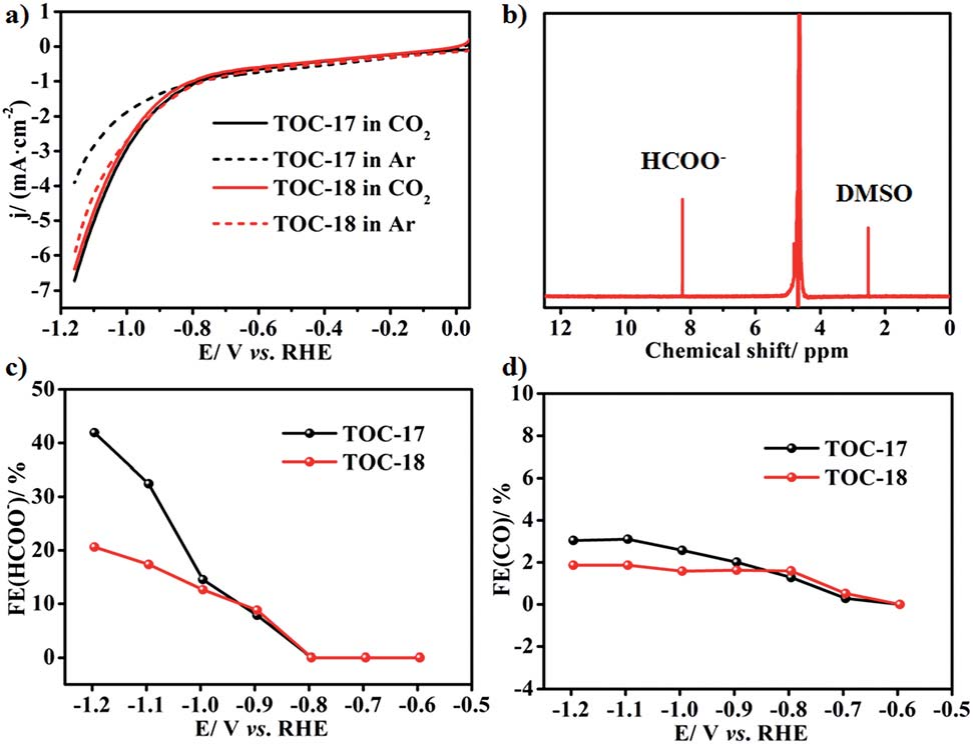
(a) Linear sweep voltammetry (LSV) of **TOC-17** and **TOC-18** in Ar or CO_2_ saturated 0.5 M KHCO_3_ solution. (b) ^1^H NMR spectrum of the KHCO_3_ catholyte after 3600 s of CO_2_ reduction on the **TOC-17** derived electrode, *E*(RHE) = –1.096 V. (c) and (d) The faradaic efficiencies (FE) of formate and CO for **TOC-17** and **TOC-18** derived electrodes at various potentials.

Recently, SnO_2_ has shown interesting energy conversion applications through electrocatalytically reducing CO_2_ to formate.^2^ Considering the similar layered characteristics of the **Sn_26_** cluster core and rutile SnO_2_, the obtained **Sn_26_** may also have potential application in the electrocatalytic CO_2_ reduction reaction (CO_2_RR). Therefore, carbon paper with **TOC-17** modification was used as the working electrode to study its catalytic activity towards the CO_2_RR. A linear sweep voltammetry (LSV) test was conducted in Ar or CO_2_ saturated 0.5 M KHCO_3_ solution, respectively. As shown in Fig. 4a, the LSV curve of the **TOC-17** derived electrode in the CO_2_ saturated electrolyte exhibits the onset potential at approximately –0.69 V. Beyond this onset potential, the current density continuously increases and reaches 6.73 mA cm^–2^ at –1.159 V, which is obviously higher than that in the Ar saturated electrolyte. This indicates that the **TOC-17** derived electrode may possess high catalytic activity towards the CO_2_RR. In order to identify and quantify the reduction products, gas chromatography (GC) and nuclear magnetic resonance (NMR) spectroscopy were applied to analyze the gas and liquid phase products during the CO_2_RR process. The obtained results indicate that formate was the only liquid CO_2_RR product, with the highest faradaic efficiency (FE) of 41.90% at –1.196 V (Fig. 4). Meanwhile, only a small amount of CO was detected in the gas phase products. Powder X-ray diffraction analysis further confirmed that the structures of **TOC-17** remained rather intact after electrolysis (Fig. S46†). For comparison, the CO_2_RR application of the cage-dimer structure **TOC-18** was also studied. Although presenting higher nuclearity and also producing formate as the main product, the activity of the **TOC-18** derived electrode was significantly lower than that modified with **TOC-17**. Therefore, these results indicate that the layered Sn–O structures might be beneficial for electrocatalytic CO_2_RR applications.

## Conclusions

In summary, we have successfully synthesized a series of unprecedented high-nuclearity tin-oxo clusters by solvent dependent synthetic strategies. These obtained high-nuclearity TOCs, with core sizes ranging from 1.6 to 2.6 nm, possess a much higher number of Sn atoms (26, 34) than previously known ones (≤14). Moreover, new structural types of layered nanorods and cage-dimers were also prepared. The applied solvent environments and Sn sources have proven to play crucial roles in the assembly of these high-nuclearity tin-oxo clusters. The introduction of water into alcohol greatly increased the cluster nuclearity; the incorporation of inorganic Sn ions significantly increased the yields of layered structures, while the application of aprotic CH_3_CN produced the largest **Sn_34_** to date. Moreover, electrocatalytic CO_2_ reduction studies confirmed that the electrodes derived from the layered **Sn_26_** cluster presented better performance than those derived from the **Sn_34_** cage-dimer. Therefore, these results afford effective synthetic strategies for the assembly of high-nuclearity TOCs and also extended their potential applications.

## Conflicts of interest

There are no conflicts to declare.

## Supplementary Material

Supplementary information

Crystal structure data
