# Supplementary material for: Assembly of high-nuclearity Sn26, Sn34-oxo clusters: solvent strategies and inorganic Sn incorporation
Source: Chem Sci. 2019 Aug 12;10(39):9125–9. doi: 10.1039/c9sc02503k (PMC6855196; doi:10.1039/c9sc02503k)
Supplement: Supplementary file 1 [file SC-010-C9SC02503K-s001.pdf]

Electronic Supplementary Material (ESI) for Chemical Science.
This journal is © The Royal Society of Chemistry 2019

Supplementary Information

Assembly of High-Nuclearity Sn_{26}, Sn_{34}-Oxo Clusters: Solvent Strategy and Inorganic Sn Incoporation

Yu Zhu, Lei Zhang* and Jian Zhang

† State Key Laboratory of Structural Chemistry, Fujian Institute of Research on the Structure of Matter, Chinese Academy of Sciences, Fuzhou, Fujian 350002, P. R. China.

E-mails: LZhang@fjirsm.ac.cn

Experimental Section

Materials and Instrumentation.

All the chemical reagents were commercially purchased and used without further purification. The synthesis experiments of the tin-oxo clusters were conducted in the fume cupboard. The gloves and mask must be wear during the synthesis experiment. IR spectra were obtained on a Vertex 7.0 spectrometer with pressed KBr pellets in the range of 4000-400 cm^{-1}. Powder XRD patterns were obtained by using a Philips X'Pert-MPD diffractometer with CuK_{α} radiation (λ = 1.54056 Å). The optical diffuse reflectance spectra were measured at room temperature using a Perkin-Elmer Lambda 950 UV-vis spectrophotometer equipped with an integrating sphere attachment and $BaSO_4$ as reference. Elemental analyses (C, H and N) were performed on a vario MICRO elemental analyzer. Thermogravimetric analyses were performed on a Mettler Toledo TGA/SDTA 851^e thermal analyzer in flowing N_2 atmosphere with a heating rate of 10 ^oC/min. The energy dispersive spectroscopy (EDS) analyses of single crystals were performed on a JEOL JSM6700F field-emission scanning electron microscope equipped with a Oxford INCA system. ESI-MS was carried out on Impact II UHR-TOF (Bruker). The ^1H NMR experiments were carried on a JNM-ECZ400S spectrometer at frequency of 400 MHz. Gas chromatography (GC) was performed with an GC-2014C (SHIMADZU) gas chromatography system equipped with flame ionization detectors and a thermal conductivity detector (TCD).

Synthesis of TOC-12: Butyltin hydroxide oxide (208.8 mg, 1.0 mmol), 2,6-pyridinedicarboxylic acid (85.0 mg, 0.5 mmol), sodium hydroxide (5.0 mg, 0.125 mmol), methanol (3.0 mL) and isopropanol (2.0 ml) were mixed and sealed in a 20 mL vial, then transferred to a preheated oven at 80 ^oC for 5 days. After cooling to

room temperature, colorless crystals were obtained (yield: 60 % based on Sn). Anal. calcd for $Sn_6NaO_{38}C_{74}H_{109}N_6$ (%): C, 36.63; H, 4.50; N, 3.46. Found: C, 34.26; H, 4.21; N, 3.35.

Synthesis of TOC-13: Butyltin hydroxide oxide (208.8 mg, 1.0 mmol), phenylphosphonic acid (126.0 mg, 0.8 mmol), methanol (3.0 mL) and isopropanol (2.0 ml) were mixed and sealed in a 20 mL vial, then transferred to a preheated oven at 80 ^{o}C for 5 days. After cooling to room temperature, colorless crystals were obtained (yield: 63 % based on Sn). Anal. calcd for $Sn_6O_{20}P_4C_{54}H_{92}$ (%): C, 34.17; H, 4.85. Found: C, 34.24; H, 4.86.

Synthesis of TOC-14: Butyltin hydroxide oxide (208.8 mg, 1.0 mmol), 2,2-Bis(hydroxymethyl)propionic acid (134.0 mg, 1.0 mmol), methanol (4.0 mL) and water (1.0 ml) were mixed and sealed in a 20 mL vial, then transferred to a preheated oven at 80 ^{o}C for 5 days. After cooling to room temperature, colorless crystals were obtained (yield: 60 % based on Sn). Anal. calcd for $Sn_{12}O_{52}C_{78}H_{188}$ (%): C, 27.69; H, 5.56. Found: C, 28.36; H, 5.30.

Synthesis of TOC-15: Butyltin hydroxide oxide (417.6 mg, 2.0 mmol), 1H-tetrazole-1-acetic acid (128.0 mg, 1.0 mmol), nickel(II) chloride hexahydrate (118.5 mg, 0.5 mmol), methanol (3.0 mL), isopropanol (1.5 ml) and water (0.5 ml) were mixed and sealed in a 20 mL vial, then transferred to a preheated oven at 80 ^{o}C for 5 days. After cooling to room temperature, colorless crystals were obtained (yield: 2 % based on Sn). Anal. calcd for $Sn_{26}O_{74}C_{114}H_{272}N_{32}Cl_4$ (%): C, 21.04; H, 4.18; N,6.89. Found: C, 20.65; H, 4.00; N, 7.27.

Synthesis of TOC-16: Butyltin hydroxide oxide (208.8 mg, 1.0 mmol), isonicotinic acid N-oxide (139.0 mg, 1.0 mmol), methanol (3.0 mL), isopropanol (1.5 ml) and water (0.5 ml) were mixed and sealed in a 20 mL vial, then transferred to a preheated oven at 80 ^{o}C for 5 days. After cooling to room temperature, colorless crystals were obtained (yield: 4 % based on Sn). Anal. calcd for $Sn_{26}O_{94}C_{166}H_{304}N_{12}$ (%): C, 28.24; H, 4.31; N, 2.38. Found: C, 28.34; H, 4.50; N, 1.83.

Synthesis of TOC-17: Butyltin hydroxide oxide (208.8 mg, 1.0 mmol), isonicotinic acid N-oxide (139.0 mg, 1.0 mmol), tin chloride pentahydrate (35.0 mg, 0.1 mmol), methanol (3.0 mL), isopropanol (1.5 ml) and water (0.5 ml) were mixed and sealed in a 20 mL vial, then transferred to a preheated oven at 80 ^{o}C for 5 days. After cooling to room temperature, colorless crystals were obtained (yield: 60 % based on Sn). Anal. calcd for $Sn_{30}O_{92}C_{158}H_{340}N_{8}Cl_{12}$ (%): C, 24.28; H, 4.35; N, 1.43. Found: C, 23.97; H, 4.17; N, 1.32.

Synthesis of TOC-18: Butyltin hydroxide oxide (208.8 mg, 1.0 mmol), 2-picolinic acid (123.0 mg, 1.0 mmol), sodium hydroxide (10.0 mg, 0.250 mmol), Acetonitrile (5.0 mL) were mixed and sealed in a 20 mL vial, then transferred to a preheated oven at 80 ^{o}C for 5 days. After cooling to room temperature, colorless crystals were obtained (yield: 45 % based on Sn). Anal. calcd for $Sn_{34}Na_{2}O_{82}C_{196}H_{376}N_{10}$ (%): C, 28.47; H, 4.55; N, 1.69. Found: C, 28.56; H, 4.28; N, 1.41.

Electrochemical measurements: Electrochemical experiments were performed on a CHI 760e electrochemical workstation (Chenhua, Shanghai, China) using a gas-tight

two-compartment electrochemical cell with a Nafion-117 proton exchange membrane as the separator. Each compartment contained 12 mL of 0.5 M $KHCO_3$ electrolyte, and the electrolyte was pre-saturated with high-purity Ar or CO_2 (Ar: pH = 8.74; CO_2: pH = 7.50). The platinum net (1.0 ×1.0 cm^2) electrode and the Ag/AgCl electrode (the saturated KCl filling solution) were used as counter and reference electrode, respectively. The reference electrode potentials were converted to the value versus RHE by the equation: E (*vs*. RHE) = E (*vs*. Ag/AgCl) + 0.197 V + 0.0591 V × pH. The working electrode was prepared by pipetting the 50 uL of sample ink onto a carbon paper electrode (1×1 cm^2) with a loading of 0.53 mg/cm^2. Typically, 5.3 mg of sample was dispersed into H_2O/ ethanol (370/ 80 uL) solution followed by adding 50 uL Nafion, then the mixture was ultrasonicated for 20 min to achieve a homogeneous ink.

For CO_2 electroreduction reaction, a flow of 20 sccm of CO_2 was continuously bubbled into the electrolyte to maintain its saturation. The linear sweep voltammetry (LSV) was performed at a scan rate of 5 mV/s. The electrolysis was conducted at selected potentials for 1 h to determine the reduction products and their Faradaic efficiencies.

Product analysis of CO_2 electroreduction:

The liquid products were quantified by nuclear magnetic resonance (NMR) spectroscopy at the end of each electrolysis. 10.0 mL of D_2O was mixed with 3.53 μL of dimethyl sulfoxide (DMSO) as solution A for next step. Then, 500 μL of the electrolyte after electrolysis was mixed with 100 μL of D_2O and 50 μL of solution A (DMSO as internal standard) for ^{1}H NMR analysis. The water suppression method was used. The peak was quantified by the integral area ratio of the reduction product

formate to DMSO. The concentration of formate was obtained using the calibration curve shown in Figure S44. The calibration curve was made by measuring standard solutions of formate.

The gaseous products (H_2 and CO) were periodically sampled and examined by gas chromatography (GC-2014C, SHIMADZU) with N_2 as the carrier gas. They were first analyzed by a thermal conductivity detector (TCD) for the H_2 concentration, and then analyzed by flame ionization detector (FID) with a methanizer for CO. The concentration of gaseous products was quantified by the integral area ratio of the reduction products to standards.

The faradic efficiency of formate was calculated as follow:

$$FE(\%) = \frac{Q_{formate}}{Q_{total}} = \frac{n_{formate} \times N \times F \times 100\ \%}{j \times t} \qquad (1)$$

Where $n_{formate}$ is the measured amount of formate in the cathodic compartment; N is the number of electrons required to form a molecule of formate (N = 2); F is the Faraday constant; j is the recorded current; t is the reaction time.

The faradic efficiencies of gaseous products were calculated as follow[1]:

The volume of the sample loop (V_0) in GC is 1 cm^3 and the flow rate of the gas is v = 20 cm^3/ min. The time it takes to fill the sample loop is:

$$t_0 = \frac{V_0}{v} = \frac{1\ cm^3}{20\ cm^3/min} = 0.05\ min = 3\ s \qquad (2)$$

According to the ideal gas law, under ambient temperature of 25 ^oC, the amount of gas in each vial (V_0 = 1 cm^3) is:

$$n = \frac{P \times V_0}{R \times T_0} = \frac{1.013 \times 10^5\ Pa \times 1 \times 10^{-6}\ m^3}{8.314\ J{\cdot}K^{-1}{\cdot}mol^{-1} \times 298.15\ K} = 4.0866 \times 10^{-5}\ mol \qquad (3)$$

The number of electrons required to form a molecule of CO or H_2 are 2. Therefore, the number of electrons (n_i) needed to get x_i ppm of CO or H_2 is:

$$n_i = x_i \times n \times N_A \times 2 \qquad (4)$$

Total number of electrons (n_{total}) measured during this sampling period:

$$n_{total} = \frac{j \times t_0}{e} \qquad (5)$$

The Faraday constant F is:

$$F = N_A \times e = 6.022 \times 10^{23}\ mol^{-1} \times 1.6022 \times 10^{-19}\ C = 96484.484\ C{\cdot}mol^{-1} \qquad (6)$$

Hence, the faradic efficiency of CO or H_2 is

$$\mathrm{FE}(\%) = \frac{n_i}{n_{total}} \times 100\ \% = \frac{x_i \times n \times F \times 2}{I_0 \times t_0} \times 100\ \% \qquad (7)$$

Where i represents CO or H_2; I_0 is the recorded current obtained from the chronoamperogram; N_A is the Avogadro constant; e is elementary charge.

X-ray Crystallography:

X-ray diffraction data of compounds **TOC-12**, **TOC-13** and **TOC-14** were collected on a SuperNova (Dual, Cu at zero, Atlas) diffractometer with graphite-monochromated CuK_α (λ = 1.54184 Å) radiation. X-ray diffraction data of compounds **TOC-16**, **TOC-17** and **TOC-18** were collected on a MM007-Saturn724+ diffractometer with graphite-monochromated MoK_α (λ = 0.71073 Å) radiation. Crystal-structure determination of compound **TOC-15** by X-ray diffraction was performed on a UltraX-Saturn724+ diffractometer with graphite-monochromated MoK_α (λ = 0.71073 Å). The program SADABS was used for absorption correction. The structures were solved by direct methods and refined on F^2 by full-matrix least-

squares methods with the SHELX 2016 program package.[2] The disordered solvent molecules were removed using the SQUEEZE routine of PLATON.[3]

Table S1. Crystal data collection and refinement details for compounds.

Compound	**TOC-12**	**TOC-13**	**TOC-14**	**TOC-15**
CCDC No.	1563679	1563680	1895007	1563685
Formula	$Sn_6NaO_{38}C_{74}H_{109}N_6$	$Sn_6O_{20}P_4C_{54}H_{92}$	$Sn_{12}O_{52}C_{78}H_{188}$	$Sn_{26}O_{74}N_{32}Cl_4C_{114}H_{272}$
Fw	2425.80	1897.29	3382.55	6503.36
Crystal system	Monoclinic	Monoclinic	Monoclinic	Triclinic
space group	*P*2(1)/*n*	*C*2/*c*	*P*2(1)/*n*	*P*-1
a/ Å	12.9255(2)	26.5875(5)	17.8261(15)	16.154(13)
b/ Å	19.9189(2)	12.3043(2)	20.7264(8)	18.360(16)
c/ Å	18.8428(3)	22.3663(4)	19.1980(15)	23.56(2)
α/deg.	90	90	90	73.021(8)
β/deg	95.4880(10)	98.407(2)	115.285(10)	89.189(10)
γ/deg	90	90	90	68.918(11)
V/ $Å^3$	4829.06(12)	7238.3(2)	6413.5(9)	6204(9)
Z	2	4	2	1
Dc /g cm^{-3}	1.668	1.741	1.752	1.741
F(000)	2420	3744	3344	3140
T /K	297.25(10)	296.76(10)	100.02(13)	293(2)
μ /mm^{-1}	12.924	17.580	18.902	2.677
θ range / °	4.555-74.552	3.966-74.514	3.474-62.492	2.502- 27.331
Reflections collected	27808	24697	19839	69435
Independent reflections	9410	7284	9846	27189
GOF[a] (F^2)	1.016	1.016	1.010	1.137
R_1/wR_2 [$I > 2\sigma(I)$]	0.0652/ 0.1439	0.0406/ 0.1046	0.0997/ 0.1984	0.0873/ 0.1262
R_1/wR_2[b] (all data)	0.0769/ 0.1518	0.0443/ 0.1098	0.1412/ 0.2143	0.1443/ 0.1425

Compound	**TOC-16**	**TOC-17**	**TOC-18**
CCDC No.	1895008	1895009	1895010
Formula	$Sn_{26}O_{94}C_{166}H_{304}N_{12}$	$Sn_{30}O_{92}C_{158}H_{340}N_8Cl_{12}$	$Sn_{34}Na_2O_{82}C_{196}H_{376}N_{10}$
Fw	7058.13	7810.46	8266.49
Crystal system	Triclinic	Triclinic	Monoclinic
space group	*P*-1	*P*-1	*P*2(1)/*n*
a/ Å	16.080(3)	16.627(3)	29.065(6)

b/ Å	16.408(3)	19.217(4)	17.858(3)
c/ Å	26.664(5)	24.646(6)	57.743(11)
α/deg.	99.647(2)	87.755(8)	90
β/deg	94.408(3)	84.536(9)	101.046(3)
γ/deg	105.134(2)	65.502(6)	90
V/ $Å^3$	6641(2)	7133(3)	29415(9)
Z	1	1	4
Dc /g cm^{-3}	1.765	1.818	1.867
F(000)	3436	3784	16000
T /K	293(2)	293(2)	293(2)
μ /mm^{-1}	2.473	2.756	2.901
θ range / °	2.389-27.488	2.028-27.299	2.061- 27.483
Reflections collected	70960	55917	199801
Independent reflections	30189	30324	66522
GOF[a] (F^2)	1.030	1.080	1.174
R_1/wR_2 [$I > 2\sigma(I)$]	0.0493 / 0.0919	0.0768/ 0.1458	0.1124 / 0.1679
R_1/wR_2[b] (all data)	0.0677 / 0.0980	0.1033/ 0.1571	0.1503 / 0.1830

^{a}GOF = $[\Sigma w(Fo^2–Fc^2)^2/(n_{obc}–n_{param})]^{1/2}$. $^bR_1 = ||Fo||Fc||/\Sigma|Fo|$, $wR_2 = [\Sigma w(Fo^2–Fc^2)^2/\Sigma w(Fo^2)^2]^{1/2}$.

Table S2. Selected bond lengths (Å) for **TOC-12**.

Bond	Length(Å)	Bond	Length(Å)
Sn(1)-O(3)	2.033(5)	Sn(3)-O(2)	2.021(5)
Sn(1)-C(1)	2.143(10)	Sn(3)-C(9)	2.143(9)
Sn(1)-O(1)	2.219(5)	Sn(3)-O(3)	2.217(5)
Sn(1)-O(5)	2.238(6)	Sn(3)-O(16)	2.238(5)
Sn(1)-O(8)	2.245(6)	Sn(3)-O(13)	2.243(7)
Sn(1)-N(1)	2.278(7)	Sn(3)-N(3)	2.270(7)
Sn(1)-O(4)	2.324(6)	Sn(3)-O(4)	2.298(5)
Sn(2)-O(1)	2.014(5)	Na(1)-O(2)#1	2.506(6)
Sn(2)-C(5)	2.123(10)	Na(1)-O(2)	2.506(6)
Sn(2)-O(2)	2.210(5)	Na(1)-O(1)	2.576(5)
Sn(2)-O(12)	2.233(6)	Na(1)-O(1)#1	2.576(5)
Sn(2)-O(9)	2.234(6)	Na(1)-O(3)	2.576(6)
Sn(2)-N(2)	2.251(8)	Na(1)-O(3)#1	2.576(6)
Sn(2)-O(4)	2.356(6)		

Symmetry transformations used to generate equivalent atoms: #1 -x+1, -y+1, -z+1.

Table S3. Selected bond lengths (Å) for **TOC-13**.

Bond	Length(Å)	Bond	Length(Å)
Sn(1)-O(4)	2.048(3)	Sn(2)-C(5)	2.138(5)
Sn(1)-O(10)#1	2.069(3)	Sn(2)-O(1)	2.148(3)
Sn(1)-O(5)	2.084(3)	Sn(2)-O(2)	2.184(3)
Sn(1)-C(1)	2.133(5)	Sn(3)-O(9)	2.080(3)
Sn(1)-O(1)	2.152(3)	Sn(3)-O(6)	2.090(3)
Sn(1)-O(3)	2.178(3)	Sn(3)-O(4)	2.092(3)
Sn(2)-O(4)	2.053(3)	Sn(3)-O(2)	2.134(3)
Sn(2)-O(8)	2.077(3)	Sn(3)-O(3)	2.136(3)
Sn(2)-O(7)#1	2.078(3)	Sn(3)-C(9)	2.141(5)

Symmetry transformations used to generate equivalent atoms: #1 -x+1, -y+1, -z+1.

Table S4. Selected bond lengths (Å) for **TOC-14**.

Bond	Length(Å)	Bond	Length(Å)
Sn(1)-O(9)#1	2.031(10)	Sn(4)-O(7)	2.072(11)
Sn(1)-O(9)	2.085(11)	Sn(4)-O(8)	2.105(11)
Sn(1)-O(10)	2.126(12)	Sn(4)-C(13)	2.12(2)
Sn(1)-C(1)	2.134(15)	Sn(4)-O(6)	2.128(12)
Sn(1)-O(20)	2.159(10)	Sn(4)-O(5)#1	2.153(13)
Sn(1)-O(8)	2.163(11)	Sn(4)-O(11)	2.163(14)
Sn(2)-O(20)	2.093(11)	Sn(5)-O(2)	2.100(12)
Sn(2)-O(19)	2.099(11)	Sn(5)-O(6)#1	2.114(14)
Sn(2)-C(5)	2.110(18)	Sn(5)-O(3)	2.131(12)
Sn(2)-O(3)	2.129(12)	Sn(5)-O(5)	2.134(12)
Sn(2)-O(1)	2.130(11)	Sn(5)-O(1)	2.139(12)
Sn(2)-O(4)	2.187(12)	Sn(5)-C(17)	2.146(16)
Sn(3)-O(10)	2.087(12)	Sn(6)-O(1)	2.073(11)
Sn(3)-O(7)	2.087(11)	Sn(6)-O(22)	2.081(12)
Sn(3)-O(19)	2.091(10)	Sn(6)-C(21)	2.108(16)

Sn(3)-O(9)	2.102(10)	Sn(6)-O(21)	2.115(12)
Sn(3)-O(15)	2.150(13)	Sn(6)-O(2)	2.147(12)
Sn(3)-C(9)	2.16(3)	Sn(6)-O(4)	2.204(12)

Symmetry transformations used to generate equivalent atoms: #1 -x+1, -y+1, -z+1.

Table S5. Selected bond lengths (Å) for **TOC-15**.

Bond	Length(Å)	Bond	Length(Å)
Sn(1)-O(16)	2.101(7)	Sn(7)-C(25)	2.195(12)
Sn(1)-O(14)	2.107(7)	Sn(7)-O(12)#1	2.191(6)
Sn(1)-O(11)	2.159(6)	Sn(7)-O(18)	2.238(7)
Sn(1)-O(21)	2.161(7)	Sn(8)-O(36)	2.106(7)
Sn(1)-C(1)	2.178(10)	Sn(8)-O(23)	2.119(8)
Sn(1)-O(7)	2.245(7)	Sn(8)-O(6)	2.154(9)
Sn(2)-O(11)	2.080(6)	Sn(8)-C(29)	2.206(19)
Sn(2)-O(12)	2.092(7)	Sn(8)-O(8)	2.208(8)
Sn(2)-O(10)	2.100(6)	Sn(8)-O(32)	2.214(8)
Sn(2)-O(5)#1	2.102(6)	Sn(9)-O(14)	2.067(7)
Sn(2)-O(15)	2.101(7)	Sn(9)-O(3)	2.101(7)
Sn(2)-O(5)	2.115(6)	Sn(9)-O(27)	2.145(7)
Sn(3)-O(17)	2.040(6)	Sn(9)-O(15)	2.189(6)
Sn(3)-O(1)	2.052(7)	Sn(9)-C(33)	2.208(12)
Sn(3)-O(14)	2.061(6)	Sn(9)-O(21)	2.206(7)
Sn(3)-O(5)	2.111(6)	Sn(10)-O(25)	2.108(7)
Sn(3)-O(7)	2.129(7)	Sn(10)-O(36)	2.117(8)
Sn(3)-O(4)	2.148(7)	Sn(10)-O(29)	2.136(8)
Sn(4)-O(28)	2.109(8)	Sn(10)-O(6)	2.195(8)
Sn(4)-O(1)	2.113(6)	Sn(10)-C(37)	2.221(12)
Sn(4)-O(16)	2.134(7)	Sn(10)-O(35)	2.305(10)
Sn(4)-C(13)	2.160(15)	Sn(11)-O(28)	2.111(7)
Sn(4)-O(24)	2.191(8)	Sn(11)-O(3)	2.122(7)
Sn(4)-O(31)	2.248(8)	Sn(11)-O(16)	2.121(8)

Sn(5)-O(25)	2.110(8)	Sn(11)-C(41)	2.173(11)
Sn(5)-O(29)	2.118(7)	Sn(11)-O(9)	2.186(8)
Sn(5)-O(1)	2.126(7)	Sn(11)-O(30)	2.224(9)
Sn(5)-O(24)	2.160(7)	Sn(12)-O(3)	2.106(7)
Sn(5)-C(17)	2.229(14)	Sn(12)-O(23)	2.119(8)
Sn(5)-O(34)	2.259(9)	Sn(12)-O(9)	2.128(8)
Sn(6)-O(29)	2.091(7)	Sn(12)-C(45)	2.191(14)
Sn(6)-O(17)	2.140(6)	Sn(12)-O(8)	2.191(8)
Sn(6)-O(10)#1	2.160(6)	Sn(12)-O(33)	2.253(8)
Sn(6)-O(18)	2.159(8)	Sn(13)-O(28)	1.996(8)
Sn(6)-O(4)	2.192(7)	Sn(13)-O(25)	2.005(7)
Sn(6)-C(21)	2.193(9)	Sn(13)-O(23)	2.006(8)
Sn(7)-O(17)	2.053(7)	Sn(13)-C(49B)	2.19(2)
Sn(7)-O(36)	2.126(7)	Sn(13)-C(49)	2.19(2)
Sn(7)-O(27)	2.157(7)		

Symmetry transformations used to generate equivalent atoms: #1 -x+1, -y+1, -z.

Table S6. Selected bond lengths (Å) for **TOC-16**.

Bond	Length(Å)	Bond	Length(Å)
Sn(1)-O(3)	2.060(4)	Sn(7)-O(15)	2.100(4)
Sn(1)-O(2)	2.059(4)	Sn(7)-C(25)	2.116(7)
Sn(1)-O(1)	2.067(4)	Sn(7)-O(20)	2.130(4)
Sn(1)-O(4)	2.066(3)	Sn(7)-O(29)	2.178(6)
Sn(1)-O(5)	2.082(4)	Sn(8)-O(11)	2.025(4)
Sn(1)-O(4)#1	2.089(3)	Sn(8)-O(16)	2.104(4)
Sn(2)-O(7)	2.002(4)	Sn(8)-O(12)	2.126(4)
Sn(2)-O(11)	2.012(4)	Sn(8)-O(2)#1	2.162(4)
Sn(2)-O(14)	2.032(4)	Sn(8)-C(29)	2.171(6)
Sn(2)-O(4)	2.080(3)	Sn(8)-O(10)	2.172(4)
Sn(2)-O(9)	2.099(4)	Sn(9)-O(22)	1.969(4)
Sn(2)-O(6)	2.107(4)	Sn(9)-O(23)	1.971(5)

Sn(3)-O(17)	2.080(4)	Sn(9)-O(21)	1.988(4)
Sn(3)-O(7)	2.088(4)	Sn(9)-C(33)	2.117(9)
Sn(3)-O(8)	2.105(4)	Sn(10)-O(23)	2.063(4)
Sn(3)-O(3)	2.127(4)	Sn(10)-O(13)	2.099(4)
Sn(3)-C(9)	2.168(7)	Sn(10)-O(17)	2.106(5)
Sn(3)-O(6)	2.198(4)	Sn(10)-C(37)	2.139(8)
Sn(4)-O(23)	2.078(5)	Sn(10)-O(19)	2.150(5)
Sn(4)-O(17)	2.093(4)	Sn(10)-O(25)	2.225(5)
Sn(4)-O(14)	2.101(4)	Sn(11)-O(21)	2.065(4)
Sn(4)-O(20)	2.140(5)	Sn(11)-O(16)	2.065(4)
Sn(4)-C(13)	2.152(7)	Sn(11)-O(24)	2.102(5)
Sn(4)-O(26)	2.197(5)	Sn(11)-C(41)	2.124(8)
Sn(5)-O(15)	2.079(4)	Sn(11)-O(18)	2.168(5)
Sn(5)-O(11)	2.084(4)	Sn(11)-O(28)	2.184(5)
Sn(5)-O(10)	2.109(4)	Sn(12)-O(22)	2.067(4)
Sn(5)-O(1)#1	2.133(4)	Sn(12)-O(15)	2.097(5)
Sn(5)-C(17)	2.151(7)	Sn(12)-O(16)	2.103(4)
Sn(5)-O(9)	2.212(4)	Sn(12)-C(45)	2.126(8)
Sn(6)-O(7)	2.033(4)	Sn(12)-O(24)	2.148(5)
Sn(6)-O(13)	2.080(4)	Sn(12)-O(30)	2.252(5)
Sn(6)-O(12)	2.122(4)	Sn(13)-O(21)	2.084(5)
Sn(6)-O(5)	2.151(4)	Sn(13)-O(19)	2.097(4)
Sn(6)-C(21)	2.155(7)	Sn(13)-O(13)	2.108(4)
Sn(6)-O(8)	2.185(4)	Sn(13)-C(49)	2.153(7)
Sn(7)-O(14)	2.082(4)	Sn(13)-O(18)	2.176(5)
Sn(7)-O(22)	2.082(5)	Sn(13)-O(27)	2.230(6)

Symmetry transformations used to generate equivalent atoms: #1 -x+1, -y+1, -z.

Table S7. Selected bond lengths (Å) for **TOC-17**.

Bond	Length(Å)	Bond	Length(Å)

Sn(1)-O(21)	2.029(7)	Sn(8)-O(24)	2.203(7)
Sn(1)-O(9)	2.037(6)	Sn(8)-O(19)	2.203(6)
Sn(1)-O(11)	2.043(7)	Sn(9)-O(27)	2.080(8)
Sn(1)-O(17)	2.097(8)	Sn(9)-O(22)	2.090(7)
Sn(1)-O(7)	2.103(6)	Sn(9)-O(15)	2.114(8)
Sn(1)-O(10)	2.114(8)	Sn(9)-O(35)	2.163(7)
Sn(2)-O(14)	2.059(7)	Sn(9)-C(33)	2.204(15)
Sn(2)-O(5)	2.066(7)	Sn(9)-O(30)	2.244(9)
Sn(2)-O(19)	2.070(7)	Sn(10)-O(16)	1.962(8)
Sn(2)-O(12)	2.086(7)	Sn(10)-O(20)	1.981(8)
Sn(2)-O(7)#1	2.088(7)	Sn(10)-O(27)	1.993(7)
Sn(2)-O(7)	2.099(7)	Sn(10)-C(37B)	2.126(14)
Sn(3)-O(27)	2.090(7)	Sn(10)-C(37)	2.131(16)
Sn(3)-O(15)	2.099(8)	Sn(11)-O(31)	2.088(8)
Sn(3)-O(9)	2.119(7)	Sn(11)-O(16)	2.091(7)
Sn(3)-C(9)	2.125(14)	Sn(11)-O(8)	2.106(7)
Sn(3)-O(18)	2.166(8)	Sn(11)-C(41)	2.151(15)
Sn(3)-O(33)	2.216(8)	Sn(11)-O(32)	2.197(9)
Sn(4)-O(15)	2.087(7)	Sn(11)-O(36)	2.200(8)
Sn(4)-O(24)	2.106(8)	Sn(12)-O(8)	2.091(7)
Sn(4)-O(11)	2.119(7)	Sn(12)-O(6)	2.106(8)
Sn(4)-C(13)	2.156(13)	Sn(12)-O(20)	2.112(8)
Sn(4)-O(5)	2.159(6)	Sn(12)-O(31)	2.154(8)
Sn(4)-O(10)	2.185(8)	Sn(12)-C(45)	2.194(14)
Sn(5)-O(20)	2.064(7)	Sn(12)-O(34)	2.246(8)
Sn(5)-O(9)	2.103(7)	Sn(13)-O(16)	2.100(8)
Sn(5)-O(6)	2.124(8)	Sn(13)-O(22)	2.104(7)
Sn(5)-C(17)	2.157(13)	Sn(13)-O(35)	2.105(8)
Sn(5)-O(18)	2.162(8)	Sn(13)-C(49)	2.159(15)
Sn(5)-O(39)	2.223(8)	Sn(13)-O(32)	2.172(8)
Sn(6)-O(6)	2.094(7)	Sn(13)-O(25)	2.228(8)

Sn(6)-O(21)	2.096(7)	Sn(14)-O(45)	2.111(12)
Sn(6)-O(23)	2.122(8)	Sn(14)-O(44)	2.121(12)
Sn(6)-C(21)	2.141(14)	Sn(14)-O(65)	2.252(11)
Sn(6)-O(14)#1	2.150(6)	Sn(14)-C(85)	2.28(2)
Sn(6)-O(17)	2.202(8)	Sn(14)-C(85B)	2.30(2)
Sn(7)-O(21)	2.026(7)	Sn(14)-Cl(3)	2.408(6)
Sn(7)-O(8)	2.107(6)	Sn(14)-Cl(1)	2.451(5)
Sn(7)-O(29)	2.130(7)	Sn(15)-O(44)	2.077(12)
Sn(7)-C(25)	2.150(12)	Sn(15)-C(81)	2.11(2)
Sn(7)-O(23)	2.177(7)	Sn(15)-O(45)	2.134(13)
Sn(7)-O(12)#1	2.199(6)	Sn(15)-C(81B)	2.12(2)
Sn(8)-O(11)	2.006(8)	Sn(15)-O(47)	2.287(13)
Sn(8)-O(22)	2.117(6)	Sn(15)-Cl(2)	2.405(6)
Sn(8)-O(29)	2.134(7)	Sn(15)-Cl(4)	2.448(6)
Sn(8)-C(29)	2.143(12)		

Symmetry transformations used to generate equivalent atoms: #1 -x+1, -y+1, -z.

Table S8. Selected bond lengths (Å) for **TOC-18**.

Bond	Length(Å)	Bond	Length(Å)
Sn(1)-O(41)	2.056(8)	Sn(19)-O(9)	2.045(8)
Sn(1)-O(7)	2.079(8)	Sn(19)-O(35)	2.057(9)
Sn(1)-C(1)	2.141(15)	Sn(19)-O(18)	2.102(10)
Sn(1)-O(34)	2.118(8)	Sn(19)-C(73)	2.117(18)
Sn(1)-O(33)	2.144(9)	Sn(20)-O(26)	2.070(9)
Sn(1)-O(11)	2.183(8)	Sn(20)-O(35)	2.084(9)
Sn(2)-O(37)	2.084(9)	Sn(20)-O(24)	2.099(10)
Sn(2)-O(23)	2.087(9)	Sn(20)-O(49)	2.116(10)
Sn(2)-C(5)	2.123(15)	Sn(20)-O(47)	2.160(9)
Sn(2)-O(31)	2.113(8)	Sn(20)-C(77)	2.173(15)
Sn(2)-O(107)	2.125(9)	Sn(21)-O(37)	1.995(9)
Sn(2)-O(42)	2.190(10)	Sn(21)-O(39)	2.021(9)

Sn(3)-O(5)	2.024(8)	Sn(21)-O(57)	2.078(10)
Sn(3)-O(22)	2.086(10)	Sn(21)-O(31)	2.106(9)
Sn(3)-O(17)	2.101(9)	Sn(21)-C(81)	2.09(2)
Sn(3)-C(9)	2.156(14)	Sn(22)-O(16)	2.099(8)
Sn(3)-O(12)	2.189(10)	Sn(22)-O(36)	2.106(9)
Sn(3)-O(56)	2.201(10)	Sn(22)-O(30)	2.124(9)
Sn(4)-O(27)	2.024(9)	Sn(22)-O(9)	2.138(8)
Sn(4)-O(24)	2.035(9)	Sn(22)-O(19)	2.133(9)
Sn(4)-O(10)	2.040(8)	Sn(22)-C(85)	2.179(15)
Sn(4)-O(20)	2.097(9)	Sn(23)-O(22)	1.989(9)
Sn(4)-C(13)	2.162(18)	Sn(23)-O(30)	2.019(8)
Sn(5)-O(48)	2.084(9)	Sn(23)-O(54)	2.098(10)
Sn(5)-O(15)	2.096(9)	Sn(23)-C(89)	2.14(2)
Sn(5)-O(7)	2.114(9)	Sn(23)-O(16)	2.144(10)
Sn(5)-O(40)	2.120(10)	Sn(24)-O(32)	2.026(9)
Sn(5)-O(32)	2.133(8)	Sn(24)-O(18)	2.041(9)
Sn(5)-C(17)	2.152(16)	Sn(24)-C(93)	2.14(2)
Sn(6)-O(5)	1.991(9)	Sn(24)-O(40)	2.079(9)
Sn(6)-O(41)	2.081(8)	Sn(24)-O(52)	2.101(9)
Sn(6)-C(21)	2.128(15)	Sn(25)-O(26)	2.091(8)
Sn(6)-O(34)	2.146(9)	Sn(25)-O(15)	2.096(9)
Sn(6)-O(13)	2.185(9)	Sn(25)-O(47)	2.106(10)
Sn(7)-O(7)	2.037(8)	Sn(25)-O(27)	2.130(9)
Sn(7)-O(15)	2.067(9)	Sn(25)-O(60)	2.144(10)
Sn(7)-O(20)	2.066(9)	Sn(25)-C(97)	2.194(13)
Sn(7)-C(25)	2.10(2)	Sn(26)-O(9)	2.070(9)
Sn(7)-O(27)	2.096(9)	Sn(26)-O(10)	2.089(9)
Sn(8)-O(41)	2.074(9)	Sn(26)-O(24)	2.156(9)
Sn(8)-O(32)	2.079(8)	Sn(26)-O(35)	2.168(9)
Sn(8)-O(13)	2.130(8)	Sn(26)-C(101)	2.12(2)
Sn(8)-O(46)	2.138(10)	Sn(26)-O(36)	2.271(8)

Sn(8)-O(14)	2.178(9)	Sn(27)-O(23)	1.984(9)
Sn(8)-C(29)	2.19(2)	Sn(27)-O(53)	2.037(9)
Sn(9)-O(16)	2.111(10)	Sn(27)-O(51)	2.092(9)
Sn(9)-O(28)	2.114(10)	Sn(27)-O(21)	2.137(9)
Sn(9)-O(17)	2.129(10)	Sn(27)-C(105)	2.166(18)
Sn(9)-O(22)	2.139(9)	Sn(28)-O(13)	1.996(9)
Sn(9)-C(33)	2.162(19)	Sn(28)-O(54)	2.069(11)
Sn(9)-O(19)	2.143(8)	Sn(28)-O(56)	2.167(10)
Sn(10)-O(6)	2.045(9)	Sn(28)-O(65)	2.179(11)
Sn(10)-O(20)	2.083(9)	Sn(28)-C(109)	2.226(17)
Sn(10)-O(11)	2.107(8)	Sn(28)-N(6)	2.393(9)
Sn(10)-C(37)	2.160(18)	Sn(29)-O(57)	2.046(10)
Sn(10)-O(29)	2.167(8)	Sn(29)-O(4)	2.071(8)
Sn(10)-O(33)	2.164(9)	Sn(29)-O(43)	2.083(10)
Sn(11)-O(37)	2.098(9)	Sn(29)-O(68)	2.158(10)
Sn(11)-O(8)	2.091(9)	Sn(29)-C(113)	2.154(17)
Sn(11)-O(4)	2.104(8)	Sn(29)-N(2)	2.337(16)
Sn(11)-O(107)	2.132(8)	Sn(30)-O(38)	2.061(9)
Sn(11)-C(41)	2.174(15)	Sn(30)-O(43)	2.089(9)
Sn(11)-O(25)	2.192(9)	Sn(30)-O(62)	2.144(10)
Sn(12)-O(36)	2.067(10)	Sn(30)-O(59)	2.130(9)
Sn(12)-O(28)	2.097(9)	Sn(30)-C(117)	2.143(19)
Sn(12)-O(6)	2.121(9)	Sn(30)-O(64)	2.136(11)
Sn(12)-O(10)	2.133(8)	Sn(31)-O(38)	2.061(9)
Sn(12)-O(19)	2.143(9)	Sn(31)-O(39)	2.111(10)
Sn(12)-C(45)	2.18(2)	Sn(31)-O(62)	2.151(10)
Sn(13)-O(17)	1.979(9)	Sn(31)-O(57)	2.147(10)
Sn(13)-O(6)	2.024(8)	Sn(31)-O(44)	2.147(10)
Sn(13)-O(29)	2.097(9)	Sn(31)-C(121)	2.21(3)
Sn(13)-C(49)	2.14(2)	Sn(32)-O(38)	2.041(9)
Sn(13)-O(28)	2.137(10)	Sn(32)-O(53)	2.104(10)

Sn(14)-O(21)	2.100(9)	Sn(32)-O(51)	2.135(10)
Sn(14)-O(23)	2.103(9)	Sn(32)-O(64)	2.146(11)
Sn(14)-O(8)	2.102(8)	Sn(32)-O(44)	2.161(11)
Sn(14)-O(107)	2.125(9)	Sn(32)-C(125)	2.17(3)
Sn(14)-C(53)	2.168(16)	Sn(33)-O(51)	2.051(11)
Sn(14)-O(50)	2.214(10)	Sn(33)-O(31)	2.071(9)
Sn(15)-O(8)	1.985(8)	Sn(33)-O(39)	2.093(10)
Sn(15)-O(43)	2.046(8)	Sn(33)-C(129)	2.115(18)
Sn(15)-O(59)	2.060(9)	Sn(33)-O(66)	2.145(10)
Sn(15)-O(4)	2.111(9)	Sn(33)-N(7)	2.325(10)
Sn(15)-C(57)	2.23(2)	Sn(34)-O(59)	2.081(9)
Sn(16)-O(34)	2.027(9)	Sn(34)-O(21)	2.076(9)
Sn(16)-O(29)	2.058(9)	Sn(34)-O(53)	2.085(11)
Sn(16)-O(12)	2.168(9)	Sn(34)-O(101)	2.157(10)
Sn(16)-O(3)	2.185(10)	Sn(34)-C(133)	2.139(19)
Sn(16)-C(61)	2.148(19)	Sn(34)-N(8)	2.340(19)
Sn(16)-N(1)	2.389(9)	Na(1)-O(48)	2.241(10)
Sn(17)-O(26)	2.091(9)	Na(1)-O(14)	2.368(9)
Sn(17)-O(40)	2.085(9)	Na(1)-O(11)	2.389(10)
Sn(17)-O(60)	2.122(9)	Na(1)-O(19)	2.539(10)
Sn(17)-O(52)	2.121(8)	Na(1)-O(9)	2.581(10)
Sn(17)-O(49)	2.140(10)	Na(1)-O(10)	2.625(10)
Sn(17)-C(65)	2.152(16)	Na(2)-O(61)	2.280(12)
Sn(18)-O(30)	2.044(10)	Na(2)-O(45)	2.270(13)
Sn(18)-O(18)	2.097(9)	Na(2)-O(55)	2.289(12)
Sn(18)-C(69)	2.11(2)	Na(2)-O(107)	2.558(10)
Sn(18)-O(14)	2.118(8)	Na(2)-O(60)	2.836(12)
Sn(18)-O(46)	2.129(11)	Na(2)-O(49)	2.870(11)
Sn(18)-O(54)	2.163(9)	Na(2)-O(47)	2.920(12)
Sn(19)-O(52)	2.012(9)		

Table S9. Bond valence sum calculations[a] of μ_2-O and μ_3-O atoms in asymmetric unit of **TOC-12**.

**O1 1.297**<br>Sn1-O1 0.428 d=2.219(5)<br>Sn2-O1 0.745 d=2.014(5)<br>Na1-O1 0.124 d=2.576(5)	**O2 1.320**<br>Sn2-O2 0.439 d=2.210(5)<br>Sn3-O2 0.731 d=2.021(5)<br>Na1-O2 0.150 d=2.506(6)	**O3 1.262**<br>Sn1-O3 0.708 d=2.033(5)<br>Sn3-O3 0.430 d=2.217(5)<br>Na1-O3 0.124 d=2.576(6)
**O4 1.681**<br>Sn1-O4 0.322 d=2.324(6)<br>Sn2-O4 0.296 d=2.356(6)<br>Sn3-O4 0.346 d=2.298(5)<br>C34-O4 0.717 d=1.513(10)		

Table S10. Bond valence sum calculations[a] of μ_2-O and μ_3-O atoms in asymmetric unit of **TOC-13**.

**O1 1.954**<br>Sn1-O1 0.513 d=2.152(3)<br>Sn2-O1 0.519 d=2.148(3)<br>C13-O1 0.922 d=1.420(6)	**O2 1.977**<br>Sn2-O2 0.470 d=2.184(3)<br>Sn3-O2 0.539 d=2.134(3)<br>C14-O2 0.968 d=1.402(6)	**O3 1.811**<br>Sn1-O3 0.478 d=2.178(3)<br>Sn3-O3 0.433 d=2.215(5)<br>C15-O3 0.900 d=1.429(6)
**O4 1.952**<br>Sn1-O4 0.679 d=2.048(3)<br>Sn2-O4 0.670 d=2.053(3)<br>Sn3-O4 0.603 d=2.092(3)		

Table S11. Bond valence sum calculations[a] of μ_2-O and μ_3-O atoms in asymmetric unit of **TOC-14**.

**O1 1.710**<br>Sn2-O1 0.544 d=2.130(11)<br>Sn5-O1 0.531 d=2.139(12)<br>Sn6-O1 0.635 d=2.073(11)	**O2 1.110**<br>Sn5-O2 0.590 d=2.100(12)<br>Sn6-O2 0.520 d=2.147(12)	**O3 1.089**<br>Sn2-O3 0.546 d=2.129(12)<br>Sn5-O3 0.543 d=2.131(12)
**O5 1.051**<br>Sn4-O5 0.512 d=2.153(13)<br>Sn5-O5 0.539 d=2.134(12)	**O6 1.115**<br>Sn4-O6 0.547 d=2.128(12)<br>Sn5-O6 0.568 d=2.114(14)	**O7 1.248**<br>Sn3-O7 0.611 d=2.087(11)<br>Sn4-O7 0.637 d=2.072(11)

**O8 1.080**<br>Sn1-O8 0.498 d=2.163(11)<br>Sn4-O8 0.582 d=2.105(11)	**O9 1.913**<br>Sn1#1-O9 0.711 d=2.031(10)<br>Sn1-O9 0.615 d=2.085(11)<br>Sn3-O9 0.587 d=2.102(10)	**O10 1.161**<br>Sn1-O10 0.550 d=2.126(12)<br>Sn3-O10 0.611 d=2.087(12)
**O19 1.197**<br>Sn2-O19 0.592 d=2.099(11)<br>Sn3-O19 0.605 d=2.091(10)	**O20 1.105**<br>Sn1-O20 0.503 d=2.159(10)<br>Sn2-O20 0.602 d=2.093(11)	

Table S12. Bond valence sum calculations[a] of μ_2-O and μ_3-O atoms in asymmetric unit of **TOC-15**.

**O1 1.792**<br>Sn3-O1 0.672 d=2.052(7)<br>Sn4-O1 0.570 d=2.113(6)<br>Sn5-O1 0.550 d=2.126(7)	**O3 1.726**<br>Sn9-O3 0.589 d=2.101(7)<br>Sn11-O3 0.556 d=2.122(7)<br>Sn12-O3 0.581 d=2.106(7)	**O4 0.979**<br>Sn3-O4 0.519 d=2.148(7)<br>Sn6-O4 0.460 d=2.192(7)
**O5 1.727**<br>Sn2#1-O5 0.587 d=2.102(6)<br>Sn2-O5 0.567 d=2.115(6)<br>Sn3-O5 0.573 d=2.111(6)	**O6 0.967**<br>Sn8-O6 0.510 d=2.154(9)<br>Sn10-O6 0.457 d=2.195(8)	**O7 0.945**<br>Sn1-O7 0.399 d=2.245(7)<br>Sn3-O7 0.546 d=2.129(7)
**O8 0.903**<br>Sn8-O8 0.441 d=2.208(8)<br>Sn12-O8 0.462 d=2.191(8)	**O9 1.015**<br>Sn11-O9 0.468 d=2.186(8)<br>Sn12-O9 0.547 d=2.128(8)	**O10 1.092**<br>Sn2-O10 0.590 d=2.100(6)<br>Sn6-O10 0.502 d=2.160(6)
**O11 1.126**<br>Sn1-O11 0.503 d=2.159(6)<br>Sn2-O11 0.623 d=2.080(6)	**O12 1.065**<br>Sn2-O12 0.603 d=2.092(7)<br>Sn7-O12 0.462 d=2.191(6)	**O14 1.880**<br>Sn1-O14 0.579 d=2.107(7)<br>Sn3-O14 0.656 d=2.061(6)<br>Sn9-O14 0.645 d=2.067(7)
**O15 1.053**<br>Sn2-O15 0.589 d=2.101(7)<br>Sn9-O15 0.464 d=2.189(6)	**O16 1.686**<br>Sn1-O16 0.589 d=2.101(7)<br>Sn4-O16 0.539 d=2.134(7)<br>Sn11-O16 0.558 d=2.121(8)	**O17 1.894**<br>Sn3-O17 0.694 d=2.040(6)<br>Sn6-O17 0.530 d=2.140(6)<br>Sn7-O17 0.670 d=2.053(7)

**O18 0.910**<br>Sn6-O18 0.503 d=2.159(8)<br>Sn7-O18 0.407 d=2.238(7)	**O21 0.944**<br>Sn1-O21 0.501 d=2.161(7)<br>Sn9-O21 0.443 d=2.206(7)	**O23 1.882**<br>Sn8-O23 0.561 d=2.119(8)<br>Sn12-O23 0.561 d=2.119(8)<br>Sn13-O23 0.761 d=2.006(8)
**O24 0.964**<br>Sn4-O24 0.462 d=2.191(8)<br>Sn5-O24 0.502 d=2.160(7)	**O25 1.916**<br>Sn5-O25 0.575 d=2.110(8)<br>Sn10-O25 0.578 d=2.108(7)<br>Sn13-O25 0.763 d=2.005(7)	**O27 1.029**<br>Sn7-O27 0.506 d=2.157(7)<br>Sn9-O27 0.523 d=2.145(7)
**O28 1.931**<br>Sn4-O28 0.576 d=2.109(8)<br>Sn11-O28 0.573 d=2.111(7)<br>Sn13-O28 0.782 d=1.996(8)	**O29 1.703**<br>Sn5-O29 0.562 d=2.118(7)<br>Sn6-O29 0.605 d=2.091(7)<br>Sn10-O29 0.536 d=2.136(8)	**O36 1.695**<br>Sn7-O36 0.550 d=2.126(7)<br>Sn8-O36 0.581 d=2.106(7)<br>Sn10-O36 0.564 d=2.117(8)

Table S13. Bond valence sum calculations[a] of μ_2-O and μ_3-O atoms in asymmetric unit of **TOC-16**.

**O1 1.185**<br>Sn1-O1 0.645 d=2.067(4)<br>Sn5-O1 0.540 d=2.133(4)	**O2 1.159**<br>Sn1-O2 0.660 d=2.059(4)<br>Sn8-O2 0.499 d=2.162(4)	**O3 1.207**<br>Sn1-O3 0.658 d=2.060(4)<br>Sn3-O3 0.549 d=2.127(4)
**O5 1.134**<br>Sn1-O5 0.620 d=2.082(4)<br>Sn6-O5 0.514 d=2.151(4)	**O6 1.032**<br>Sn2-O6 0.579 d=2.107(4)<br>Sn3-O6 0.453 d=2.198(4)	**O9 1.028**<br>Sn2-O9 0.592 d=2.099(4)<br>Sn5-O9 0.436 d=2.212(4)
**O12 1.106**<br>Sn6-O12 0.556 d=2.122(4)<br>Sn8-O12 0.550 d=2.126(4)	**O10 1.062**<br>Sn5-O10 0.576 d=2.109(4)<br>Sn8-O10 0.486 d=2.172(4)	**O8 1.051**<br>Sn3-O8 0.582 d=2.105(4)<br>Sn6-O8 0.469 d=2.185(4)
**O18 0.972**<br>Sn11-O18 0.491 d=2.168(5)<br>Sn13-O18 0.481 d=2.176(5)	**O20 1.074**<br>Sn4-O20 0.530 d=2.140(5)<br>Sn7-O20 0.544 d=2.130(4)	**O24 1.106**<br>Sn11-O24 0.587 d=2.102(5)<br>Sn12-O24 0.519 d=2.148(5)

**O19 1.111**<br>Sn10-O19 0.516 d=2.150(5)<br>Sn13-O19 0.595 d=2.097(4)	**O4 1.878**<br>Sn1-O4 0.647 d=2.066(3)<br>Sn1#1-O4 0.608 d=2.089(3)<br>Sn2-O4 0.623 d=2.080(3)	**O7 2.087**<br>Sn2-O7 0.769 d=2.002(4)<br>Sn3-O7 0.610 d=2.088(4)<br>Sn6-O7 0.708 d=2.033(4)
**O11 2.088**<br>Sn2-O11 0.749 d=2.012(4)<br>Sn5-O11 0.616 d=2.084(4)<br>Sn8-O11 0.723 d=2.025(4)	**O13 1.793**<br>Sn6-O13 0.623 d=2.080(4)<br>Sn10-O13 0.592 d=2.099(4)<br>Sn13-O13 0.578 d=2.108(4)	**O17 1.806**<br>Sn3-O17 0.623 d=2.080(4)<br>Sn4-O17 0.602 d=2.093(4)<br>Sn10-O17 0.581 d=2.106(5)
**O14 1.918**<br>Sn2-O14 0.709 d=2.032(4)<br>Sn4-O14 0.589 d=2.101(4)<br>Sn7-O14 0.620 d=2.082(4)	**O16 1.819**<br>Sn8-O16 0.584 d=2.104(4)<br>Sn11-O16 0.649 d=2.065(4)<br>Sn12-O16 0.586 d=2.103(4)	**O15 1.810**<br>Sn5-O15 0.625 d=2.079(4)<br>Sn7-O15 0.590 d=2.100(4)<br>Sn12-O15 0.595 d=2.097(5)
**O21 2.064**<br>Sn9-O21 0.799 d=1.988(4)<br>Sn11-O21 0.649 d=2.065(4)<br>Sn13-O21 0.616 d=2.084(5)	**O22 2.106**<br>Sn7-O22 0.620 d=2.082(5)<br>Sn9-O22 0.841 d=1.969(4)<br>Sn12-O22 0.645 d=2.067(4)	**O23 2.116**<br>Sn4-O23 0.627 d=2.078(5)<br>Sn9-O23 0.837 d=1.971(5)<br>Sn10-O23 0.652 d=2.063(4)

Table S14. Bond valence sum calculations[a] of μ_2-O and μ_3-O atoms in asymmetric unit of **TOC-17**.

**O5 1.150**<br>Sn2-O5 0.647 d=2.066(7)<br>Sn4-O5 0.503 d=2.159(6)	**O19 1.087**<br>Sn2-O19 0.640 d=2.070(7)<br>Sn8-O19 0.447 d=2.203(6)	**O12 1.065**<br>Sn2-O12 0.613 d=2.086(7)<br>Sn7-O12 0.452 d=2.199(6)
**O14 1.176**<br>Sn2-O14 0.660 d=2.059(7)<br>Sn6-O14 0.516 d=2.150(6)	**O24 1.028**<br>Sn4-O24 0.581 d=2.106(8)<br>Sn8-O24 0.447 d=2.203(7)	**O29 1.083**<br>Sn7-O29 0.544 d=2.130(7)<br>Sn8-O29 0.539 d=2.134(7)
**O23 1.035**<br>Sn6-O23 0.556 d=2.122(8)<br>Sn7-O23 0.479 d=2.177(7)	**O17 1.043**<br>Sn1-O17 0.595 d=2.097(8)<br>Sn6-O17 0.448 d=2.202(8)	**O10 1.037**<br>Sn1-O10 0.568 d=2.114(8)<br>Sn4-O10 0.469 d=2.185(8)

**O32 0.940**<br>Sn11-O32 0.454 d=2.197(9)<br>Sn13-O32 0.486 d=2.172(8)	**O31 1.120**<br>Sn11-O31 0.610 d=2.088(8)<br>Sn12-O31 0.510 d=2.154(8)	**O35 1.080**<br>Sn9-O35 0.498 d=2.163(7)<br>Sn13-O35 0.582 d=2.105(8)
**O18 0.993**<br>Sn3-O18 0.494 d=2.166(8)<br>Sn5-O18 0.499 d=2.162(8)	**O7 1.788**<br>Sn1-O7 0.586 d=2.103(6)<br>Sn2#1-O7 0.610 d=2.088(7)<br>Sn2-O7 0.592 d=2.099(7)	**O11 2.011**<br>Sn1-O11 0.689 d=2.043(7)<br>Sn4-O11 0.561 d=2.119(7)<br>Sn8-O11 0.761 d=2.006(8)
**O21 2.033**<br>Sn1-O21 0.715 d=2.029(7)<br>Sn6-O21 0.597 d=2.096(7)<br>Sn7-O21 0.721 d=2.026(7)	**O22 1.755**<br>Sn8-O22 0.564 d=2.117(6)<br>Sn9-O22 0.607 d=2.090(7)<br>Sn13-O22 0.584 d=2.104(7)	**O9 1.847**<br>Sn1-O9 0.700 d=2.037(6)<br>Sn3-O9 0.561 d=2.119(7)<br>Sn5-O9 0.586 d=2.103(7)
**O8 1.765**<br>Sn7-O8 0.579 d=2.107(6)<br>Sn11-O8 0.581 d=2.106(7)<br>Sn12-O8 0.605 d=2.091(7)	**O6 1.734**<br>Sn6-O6 0.600 d=2.094(7)<br>Sn5-O6 0.553 d=2.124(8)<br>Sn12-O6 0.581 d=2.106(8)	**O15 1.771**<br>Sn3-O15 0.592 d=2.099(8)<br>Sn4-O15 0.611 d=2.087(7)<br>Sn9-O15 0.568 d=2.114(8)
**O16 2.052**<br>Sn10-O16 0.857 d=1.962(8)<br>Sn11-O16 0.605 d=2.091(7)<br>Sn13-O16 0.590 d=2.100(8)	**O27 2.018**<br>Sn3-O27 0.607 d=2.090(7)<br>Sn9-O27 0.623 d=2.080(8)<br>Sn10-O27 0.788 d=1.993(7)	**O20 2.037**<br>Sn5-O20 0.651 d=2.064(7)<br>Sn10-O20 0.814 d=1.981(8)<br>Sn12-O20 0.572 d=2.112(8)
**O44 1.186**<br>Sn14-O44 0.558 d=2.121(12)<br>Sn15-O44 0.628 d=2.077(12)	**O45 1.112**<br>Sn14-O45 0.573 d=2.111(12)<br>Sn15-O45 0.539 d=2.134(13)	

Table S15. Bond valence sum calculations[a] of μ_2-O and μ_3-O atoms in asymmetric unit of **TOC-18**.

**O56 0.942**<br>Sn3-O56 0.449 d=2.201(10)<br>Sn28-O56 0.493 d=2.167(10)	**O12 0.955**<br>Sn3-O12 0.464 d=2.189(10)<br>Sn16-O12 0.491 d=2.168(9)	**O17 1.954**<br>Sn3-O17 0.589 d=2.101(9)<br>Sn9-O17 0.546 d=2.129(10)<br>Sn13-O17 0.819 d=1.979(9)

**O22 1.941**<br>Sn3-O22 0.613 d=2.086(10)<br>Sn9-O22 0.531 d=2.139(9)<br>Sn23-O22 0.797 d=1.989(9)	**O16 1.689**<br>Sn9-O16 0.573 d=2.111(10)<br>Sn22-O16 0.592 d=2.099(8)<br>Sn23-O16 0.524 d=2.144(10)	**O28 1.697**<br>Sn9-O28 0.568 d=2.114(10)<br>Sn12-O28 0.595 d=2.097(9)<br>Sn13-O28 0.534 d=2.137(10)
**O34 1.802**<br>Sn1-O34 0.562 d=2.118(8)<br>Sn6-O34 0.521 d=2.146(9)<br>Sn16-O34 0.719 d=2.027(9)	**O13 1.795**<br>Sn8-O13 0.544 d=2.130(8)<br>Sn6-O13 0.469 d=2.185(9)<br>Sn28-O13 0.782 d=1.996(9)	**O29 1.749**<br>Sn10-O29 0.493 d=2.167(8)<br>Sn13-O29 0.595 d=2.097(9)<br>Sn16-O29 0.661 d=2.058(9)
**O54 1.734**<br>Sn18-O54 0.498 d=2.163(9)<br>Sn23-O54 0.594 d=2.098(10)<br>Sn28-O54 0.642 d=2.069(11)	**O6 1.968**<br>Sn10-O6 0.685 d=2.045(9)<br>Sn12-O6 0.558 d=2.121(9)<br>Sn13-O6 0.725 d=2.024(8)	**O30 1.975**<br>Sn18-O30 0.687 d=2.044(10)<br>Sn22-O30 0.553 d=2.124(9)<br>Sn23-O30 0.735 d=2.019(8)
**O11 1.256**<br>Sn1-O11 0.472 d=2.183(8)<br>Sn10-O11 0.579 d=2.107(8)<br>Na1-O11 0.205 d=2.389(10)	**O14 1.257**<br>Sn8-O14 0.478 d=2.178(9)<br>Sn18-O14 0.562 d=2.118(8)<br>Na1-O14 0.217 d=2.368(9)	**O36 1.598**<br>Sn12-O36 0.645 d=2.067(10)<br>Sn22-O36 0.581 d=2.106(9)<br>Sn26-O36 0.372 d=2.271(8)
**O20 1.860**<br>Sn4-O20 0.595 d=2.097(9)<br>Sn7-O20 0.647 d=2.066(9)<br>Sn10-O20 0.618 d=2.083(9)	**O7 1.893**<br>Sn1-O7 0.625 d=2.079(8)<br>Sn5-O7 0.568 d=2.114(9)<br>Sn7-O7 0.700 d=2.037(8)	**O32 1.886**<br>Sn5-O32 0.540 d=2.133(8)<br>Sn8-O32 0.625 d=2.079(8)<br>Sn24-O32 0.721 d=2.026(9)
**O18 1.874**<br>Sn18-O18 0.595 d=2.097(9)<br>Sn19-O18 0.587 d=2.102(10)<br>Sn24-O18 0.692 d=2.041(9)	**O27 1.866**<br>Sn4-O27 0.725 d=2.024(9)<br>Sn7-O27 0.597 d=2.096(9)<br>Sn25-O27 0.544 d=2.130(9)	**O24 1.803**<br>Sn4-O24 0.704 d=2.035(9)<br>Sn20-O24 0.592 d=2.099(10)<br>Sn26-O24 0.507 d=2.156(9)
**O15 1.839**<br>Sn5-O15 0.597 d=2.096(9)<br>Sn7-O15 0.645 d=2.067(9)<br>Sn25-O15 0.597 d=2.096(9)	**O35 1.770**<br>Sn19-O35 0.663 d=2.057(9)<br>Sn20-O35 0.616 d=2.084(9)<br>Sn26-O35 0.491 d=2.168(9)	**O40 1.799**<br>Sn5-O40 0.559 d=2.120(10)<br>Sn17-O40 0.615 d=2.085(9)<br>Sn24-O40 0.625 d=2.079(9)
**O52 1.896**<br>Sn17-O52 0.558 d=2.121(8)<br>Sn19-O52 0.749 d=2.012(9)<br>Sn24-O52 0.589 d=2.101(9)	**O26 1.850**<br>Sn17-O26 0.605 d=2.091(9)<br>Sn20-O26 0.640 d=2.070(9)<br>Sn25-O26 0.605 d=2.091(8)	**O47 1.132**<br>Sn20-O47 0.502 d=2.160(9)<br>Sn25-O47 0.581 d=2.106(10)<br>Na2-O47 0.049 d=2.920(12)

**O49 1.151**<br>Sn17-O49 0.530 d=2.140(10)<br>Sn20-O49 0.565 d=2.116(10)<br>Na2-O49 0.056 d=2.870(11)	**O60 1.141**<br>Sn17-O60 0.556 d=2.122(9)<br>Sn25-O60 0.524 d=2.144(10)<br>Na2-O60 0.061 d=2.836(12)	**O107 1.775**<br>Sn2-O107 0.552 d=2.125(9)<br>Sn11-O107 0.541 d=2.132(8)<br>Sn14-O107 0.552 d=2.125(9)<br>Na2-O107 0.130 d=2.558(10)
**O23 2.005**<br>Sn2-O23 0.611 d=2.087(9)<br>Sn14-O23 0.586 d=2.103(9)<br>Sn27-O23 0.808 d=1.984(9)	**O37 1.994**<br>Sn2-O37 0.616 d=2.084(9)<br>Sn11-O37 0.594 d=2.098(9)<br>Sn21-O37 0.784 d=1.995(9)	**O8 1.998**<br>Sn11-O8 0.605 d=2.091(9)<br>Sn14-O8 0.587 d=2.102(8)<br>Sn15-O8 0.806 d=1.985(8)
**O21 1.754**<br>Sn34-O21 0.630 d=2.076(9)<br>Sn27-O21 0.534 d=2.137(9)<br>Sn14-O21 0.590 d=2.100(9)	**O31 1.789**<br>Sn2-O31 0.570 d=2.113(8)<br>Sn21-O31 0.581 d=2.106(9)<br>Sn33-O31 0.638 d=2.071(9)	**O4 1.795**<br>Sn11-O4 0.584 d=2.104(8)<br>Sn15-O4 0.573 d=2.111(9)<br>Sn29-O4 0.638 d=2.071(8)
**O53 1.899**<br>Sn27-O53 0.700 d=2.037(9)<br>Sn32-O53 0.584 d=2.104(10)<br>Sn34-O53 0.615 d=2.085(11)	**O51 1.814**<br>Sn27-O51 0.603 d=2.092(9)<br>Sn32-O51 0.537 d=2.135(10)<br>Sn33-O51 0.674 d=2.051(11)	**O59 1.823**<br>Sn15-O59 0.658 d=2.060(9)<br>Sn30-O59 0.544 d=2.130(9)<br>Sn34-O59 0.621 d=2.081(9)
**O39 1.906**<br>Sn21-O39 0.731 d=2.021(9)<br>Sn31-O39 0.573 d=2.111(10)<br>Sn33-O39 0.602 d=2.093(10)	**O57 1.830**<br>Sn21-O57 0.627 d=2.078(10)<br>Sn29-O57 0.683 d=2.046(10)<br>Sn31-O57 0.520 d=2.147(10)	**O43 1.909**<br>Sn15-O43 0.683 d=2.046(8)<br>Sn29-O43 0.618 d=2.083(10)<br>Sn30-O43 0.608 d=2.089(9)
**O38 2.004**<br>Sn30-O38 0.656 d=2.061(9)<br>Sn31-O38 0.656 d=2.061(9)<br>Sn32-O38 0.692 d=2.041(9)	**O44 1.021**<br>Sn31-O44 0.520 d=2.147(10)<br>Sn32-O44 0.501 d=2.161(11)	**O64 1.057**<br>Sn30-O64 0.536 d=2.136(11)<br>Sn32-O64 0.521 d=2.146(11)
**O62 1.038**<br>Sn30-O62 0.524 d=2.144(10)<br>Sn31-O62 0.514 d=2.151(10)	**O19 1.729**<br>Sn9-O19 0.526 d=2.143(8)<br>Sn12-O19 0.526 d=2.143(9)<br>Sn22-O19 0.540 d=2.133(9)<br>Na1-O19 0.137 d=2.539(10)	**O33 1.021**<br>Sn1-O33 0.524 d=2.144(9)<br>Sn10-O33 0.497 d=2.164(9)
**O46 1.079**<br>Sn8-O46 0.533 d=2.138(10)<br>Sn18-O46 0.546 d=2.129(11)	**O10 1.950**<br>Sn4-O10 0.694 d=2.040(8)<br>Sn12-O10 0.540 d=2.133(8)<br>Sn26-O10 0.608 d=2.089(9)<br>Na1-O10 0.108 d=2.625(10)	**O9 1.858**<br>Sn19-O9 0.685 d=2.045(8)<br>Sn22-O9 0.533 d=2.138(8)<br>Sn26-O9 0.640 d=2.070(9)

**O5 1.518**<br>Sn3-O5 0.725 d=2.024(8)<br>Sn6-O5 0.793 d=1.991(9)	**O48 0.922**<br>Sn5-O48 0.616 d=2.084(9)<br>Na1-O48 0.306 d=2.241(10)	**O41 1.919**<br>Sn1-O41 0.665 d=2.056(8)<br>Sn6-O41 0.621 d=2.081(8)<br>Sn8-O41 0.633 d=2.074(9)

[a] $V_i = \Sigma_j v_{ij} = \Sigma_j \exp[(r_0 - r_{ij})/B]$, where r_0 is the bond-valence parameter (here r_0 = 1.905 for Sn^{IV}-O, and 1.803 for Na^{I}-O), r_{ij} is the bond length between atoms *i* and *j*; B is a constant, the "universal parameter" ~0.37 Å; v_{ij} is the valence of a bond between atoms *i* and *j*; V_i is the sum of all bond valences of the bonds formed by a given atom *i*.[4]

References

[1] D. Ren, Y. L. Deng, A. D. Handoko, C. S. Chen and S. Malkhandi, *ACS Catal*., 2015, **5**, 2814.

[2] G. M. Sheldrick, *Acta Cryst*., 2015, **C71**, 3.

[3] A. L. Spek, *Acta Cryst*., 2015, **C71**, 9.

[4] N. E. Brese and M. O' Keeffe, *Acta Cryst*., 1991, **B47**, 192.

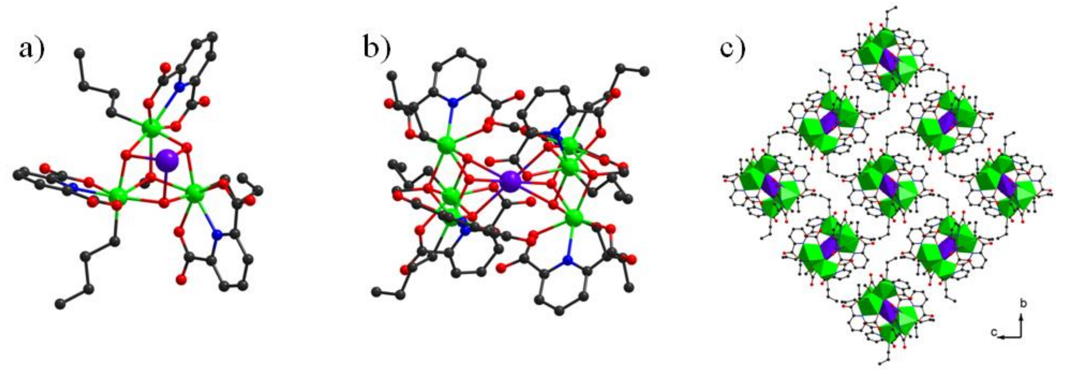

Figure S1. The asymmetric unit (a), cluster structure (b), and packing mode (c) of **TOC-12**. Atom color code: green Sn; purple Na; red O; black C; dark blue N. Polyhedral color code: green SnO_5CN; purple NaO_6.

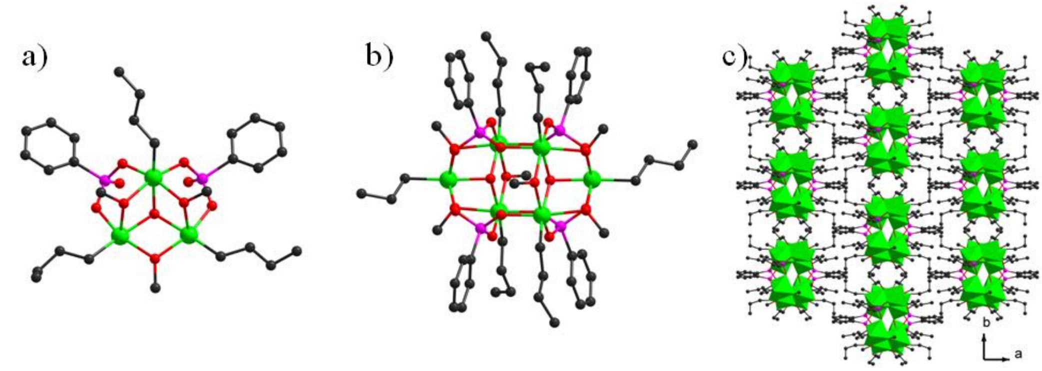

Figure S2. The asymmetric unit (a), cluster structure (b), and packing mode (c) of **TOC-13**. Atom color code: green Sn; red O; black C; purplish red P. Polyhedral color code: green SnO_5C.

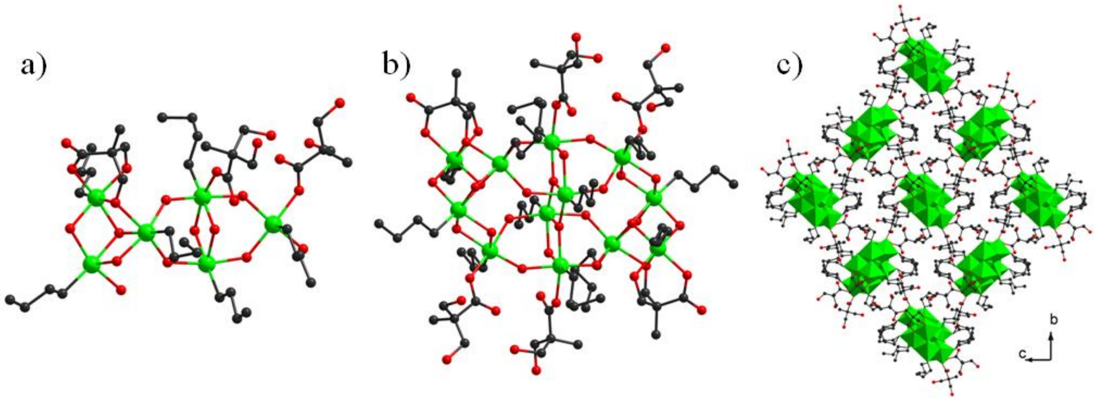

Figure S3. The asymmetric unit (a), cluster structure (b), and packing mode (c) of **TOC-14**. Atom color code: green Sn; red O; black C. Polyhedral color code: green SnO_5C.

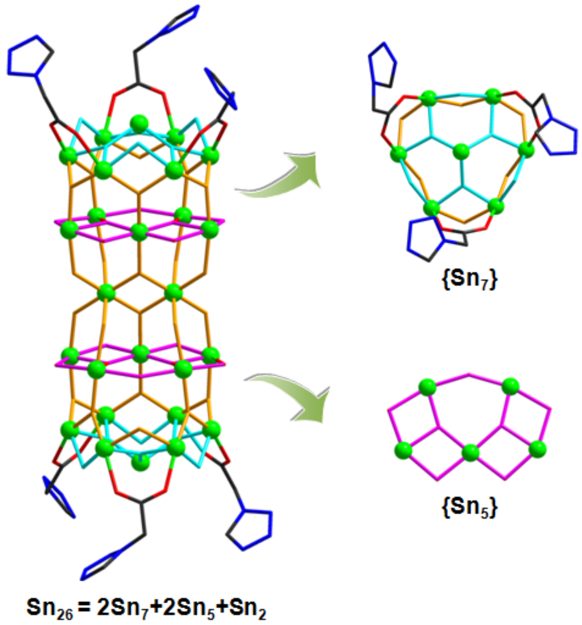

Figure S4. The ball-and-stick illustration of **Sn_{26}** cluster in **TOC-15**. Atom color code: green Sn.

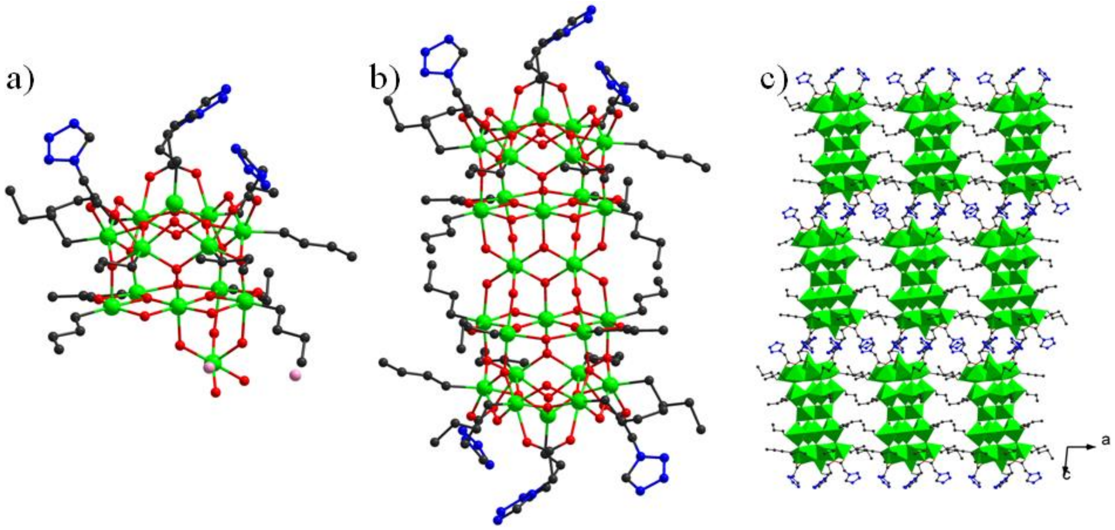

Figure S5. The asymmetric unit (a), cluster structure (b), and packing mode (c) of the **TOC-15**. Atom color code: green Sn; red O; black C; dark blue N; pink Cl. Polyhedral color code: green SnO_3C/SnO_5C/SnO_6.

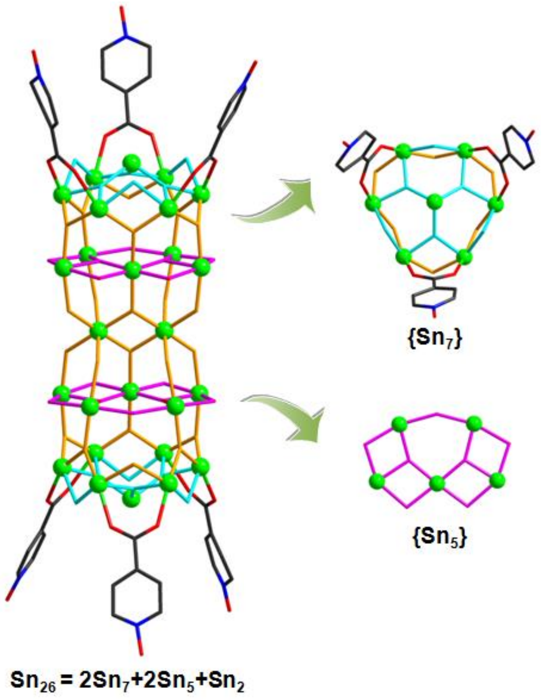

Figure S6. The ball-and-stick illustration of $\mathbf{Sn_{26}}$ cluster in **TOC-16** and **TOC-17**. Atom color code: green Sn.

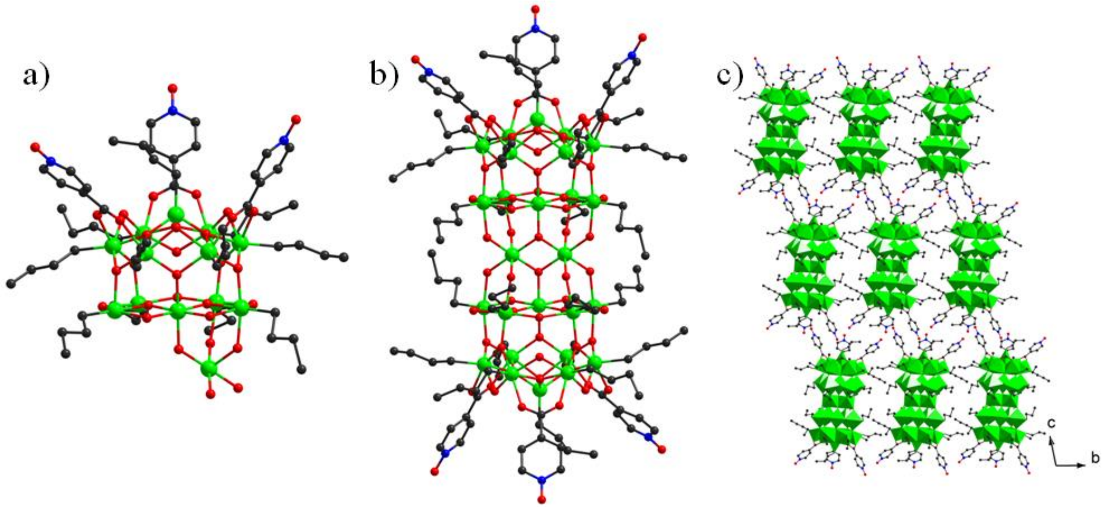

Figure S7. The asymmetric unit (a), cluster structure (b), and packing mode (c) of the **TOC-16**. Atom color code: geren Sn; red O; black C; dark blue N. Polyhedral color code: green $SnO_3C/SnO_5C/SnO_6$.

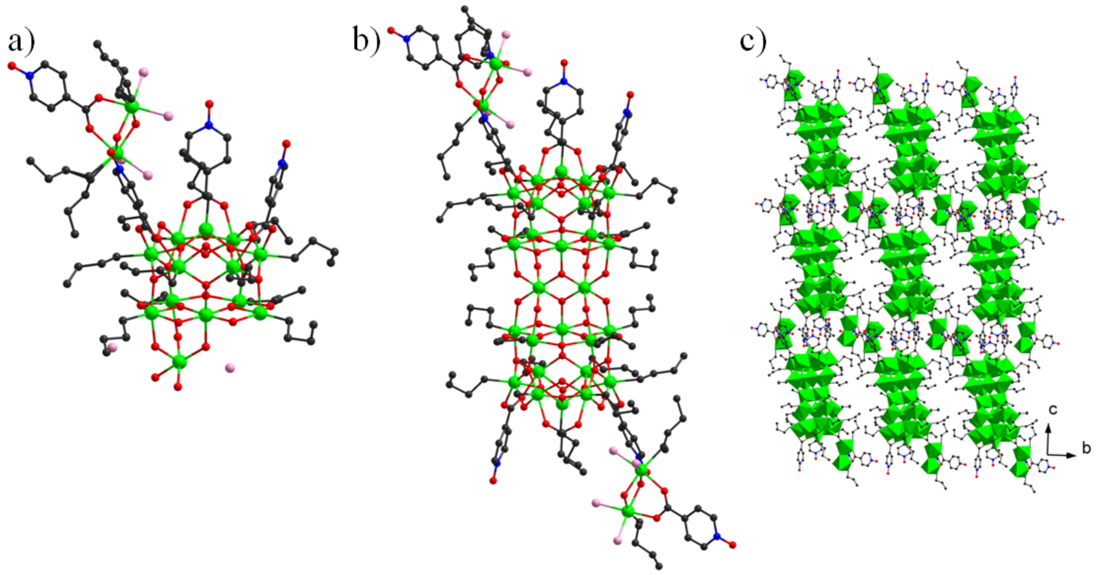

Figure S8. The asymmetric unit (a), cluster structure (b), and packing mode (c) of the **TOC-17**. Atom color code: green Sn; red O; black C; pink Cl. Polyhedral color code: green SnO_3C/ SnO_5C/SnO_6.

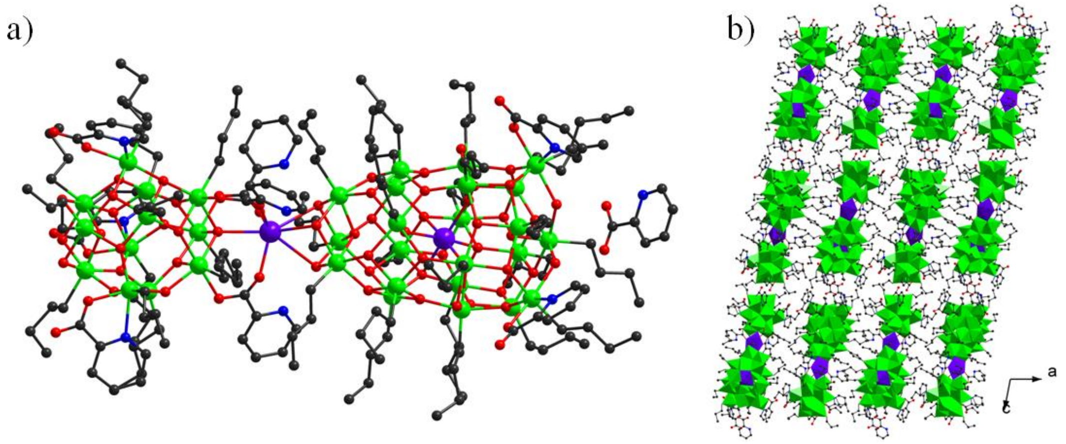

Figure S9. The asymmetric unit (a), and packing mode of the **TOC-18**. Atom color code: green Sn; purple Na; red O; black C; dark blue N. Polyhedral color code: green SnO_5C/SnO_4C/SnO_4NC; purple NaO_7/NaO_6.

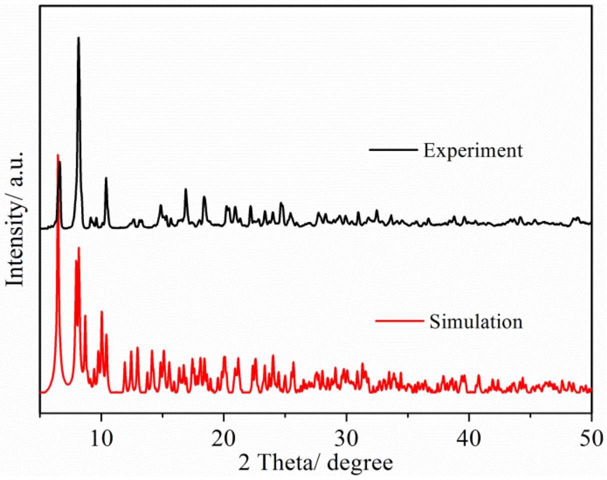

Figure S10. Simulated and experimental PXRD pattern of compound **TOC-12**.

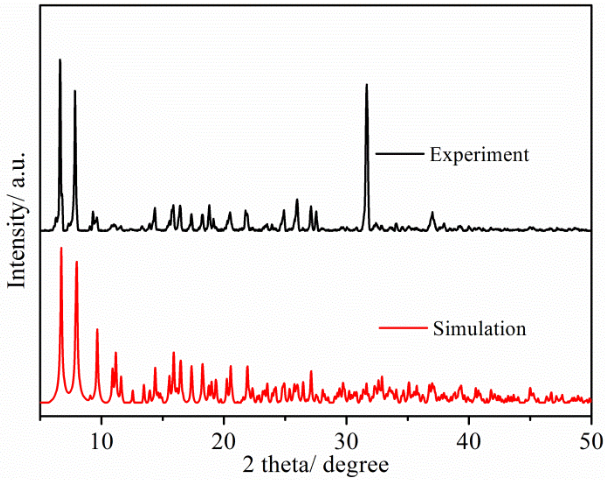

Figure S11. Simulated and experimental PXRD pattern of compound **TOC-13**.

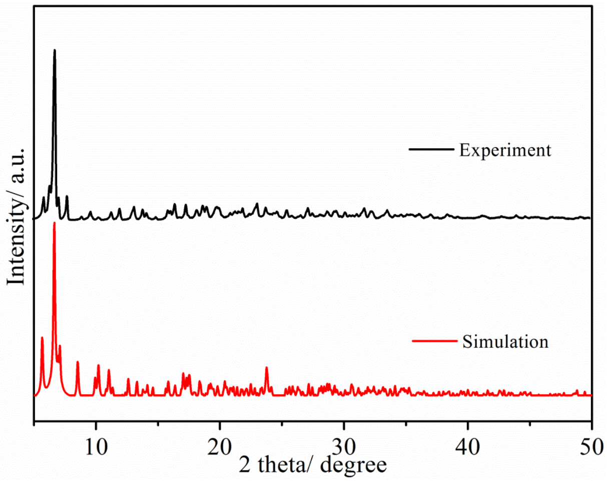

Figure S12. Simulated and experimental PXRD pattern of compound **TOC-14**.

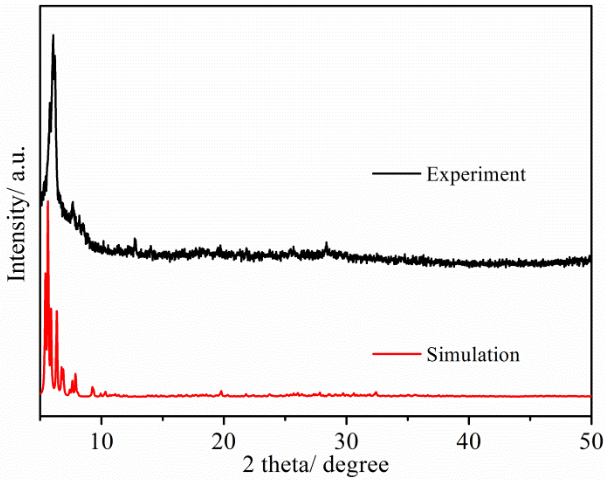

Figure S13. Simulated and experimental PXRD pattern of compound **TOC-15**.

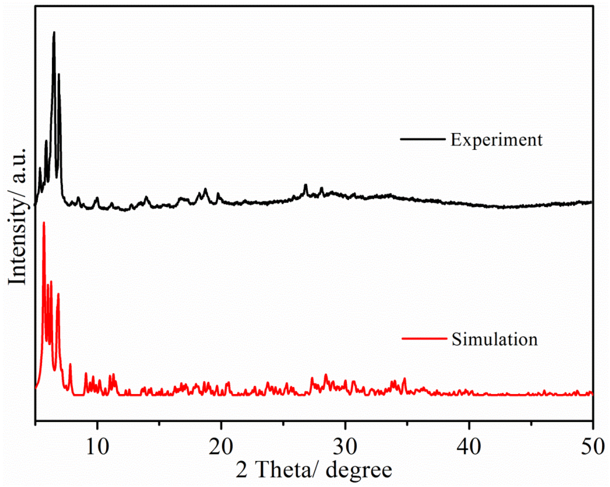

Figure S14. Simulated and experimental PXRD pattern of compound **TOC-16**.

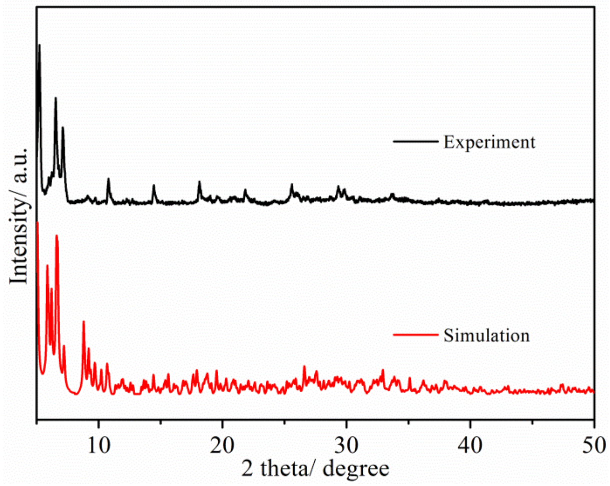

Figure S15. Simulated and experimental PXRD pattern of compound **TOC-17**.

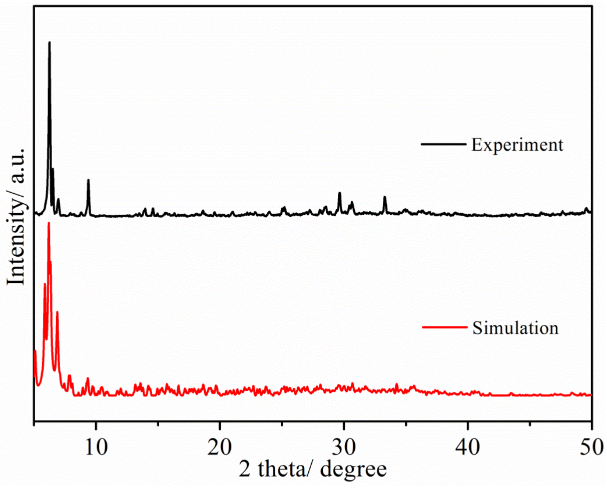

Figure S16. Simulated and experimental PXRD pattern of compound **TOC-18**.

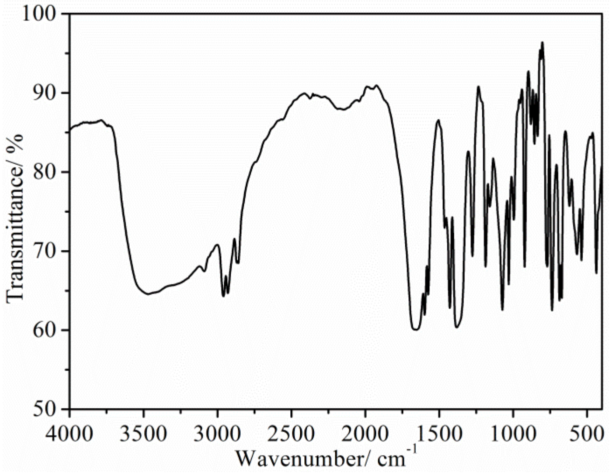

Figure S17. IR spectrum of compound **TOC-12**.

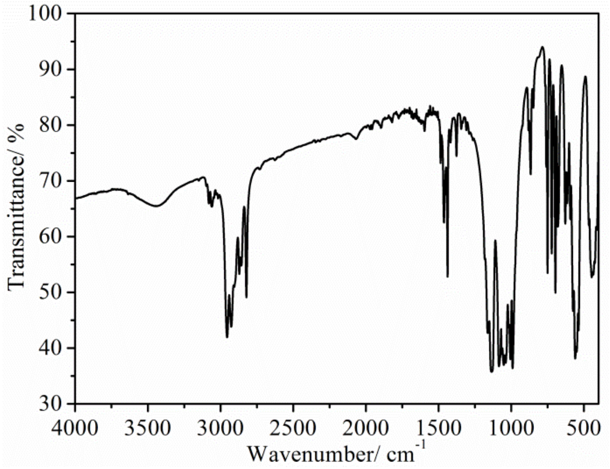

Figure S18. IR spectrum of compound **TOC-13**.

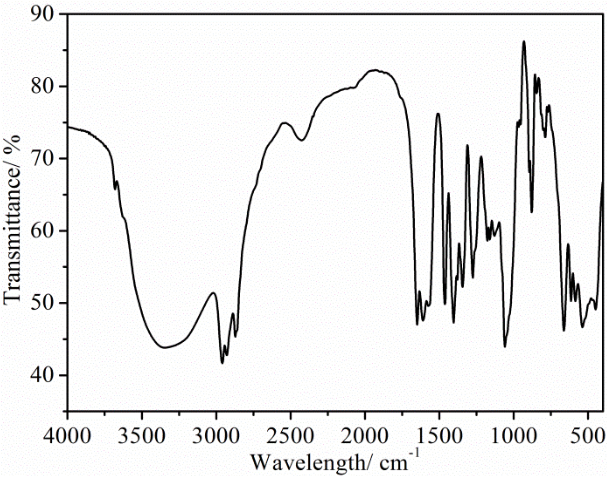

Figure S19. IR spectrum of compound **TOC-14**.

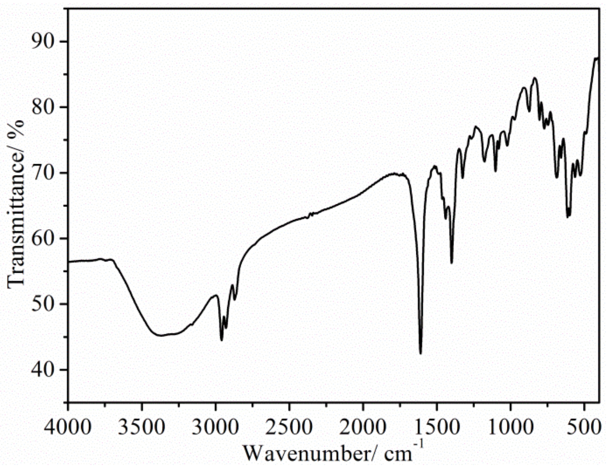

Figure S20. IR spectrum of compound **TOC-15**.

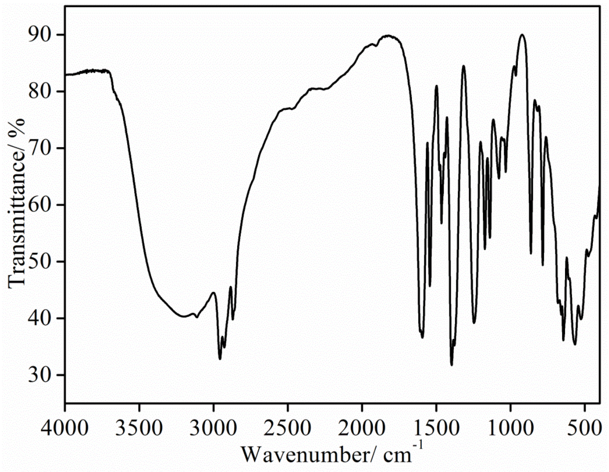

Figure S21. IR spectrum of compound **TOC-16**.

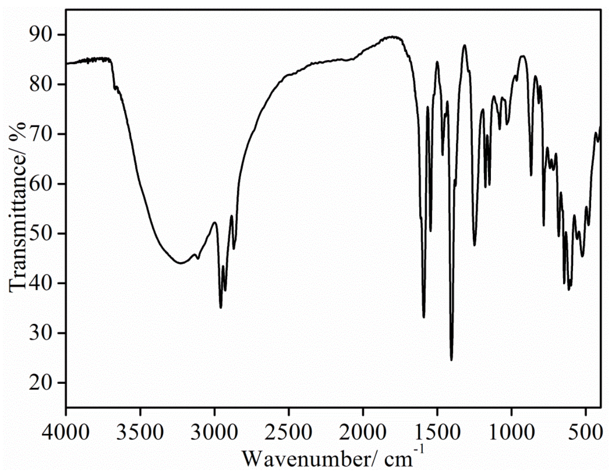

Figure S22. IR spectrum of compound **TOC-17**.

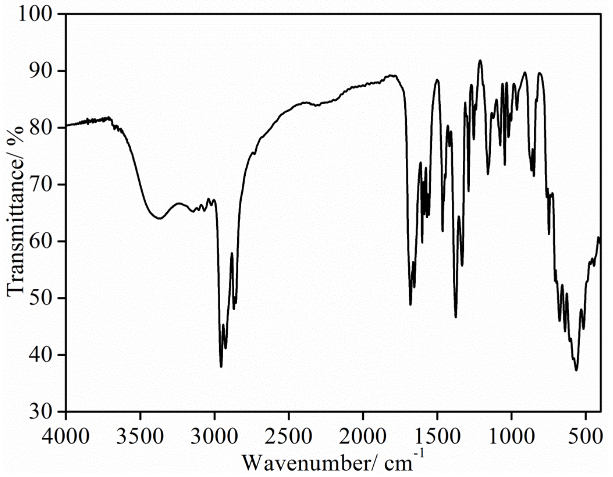

Figure S23. IR spectrum of compound **TOC-18**.

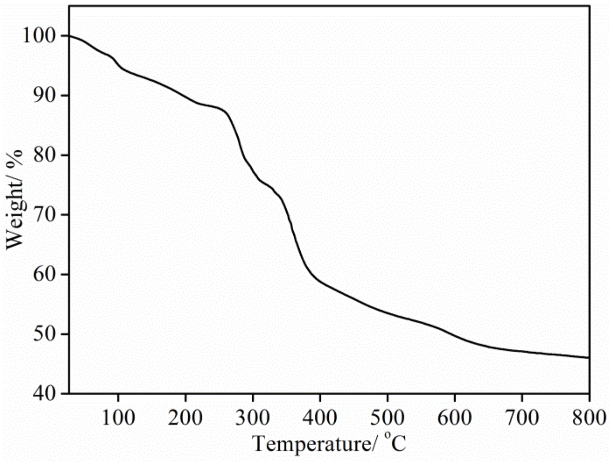

Figure S24. TG curve of compound **TOC-12** in N_2 atmosphere.

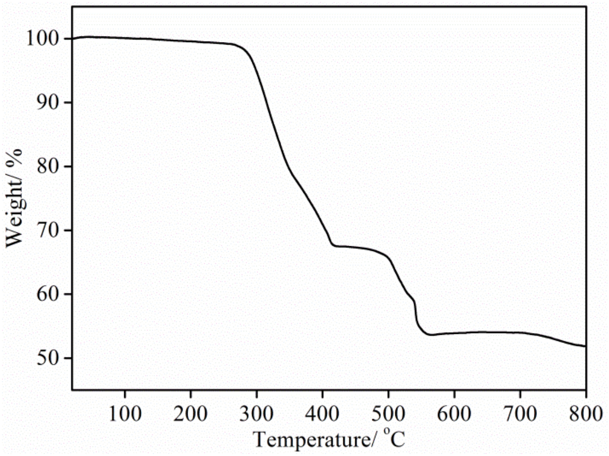

Figure S25. TG curve of compound **TOC-13** in N_2 atmosphere.

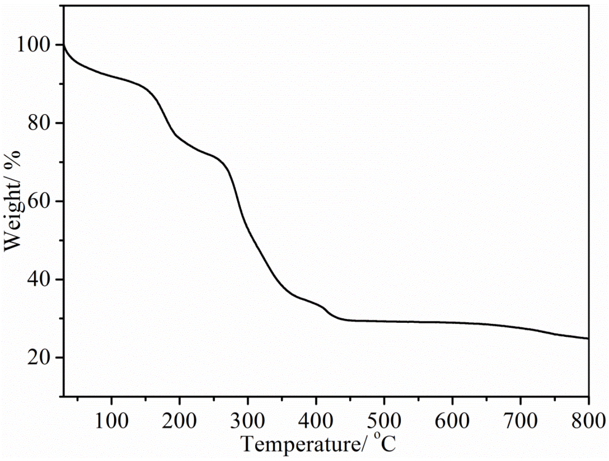

Figure S26. TG curve of compound **TOC-14** in N_2 atmosphere.

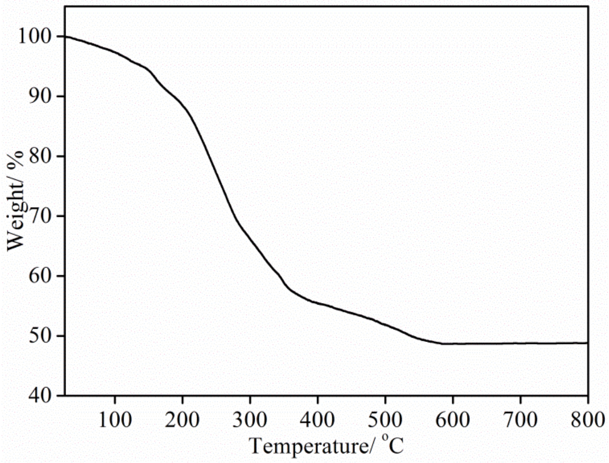

Figure S27. TG curve of compound **TOC-15** in N_2 atmosphere.

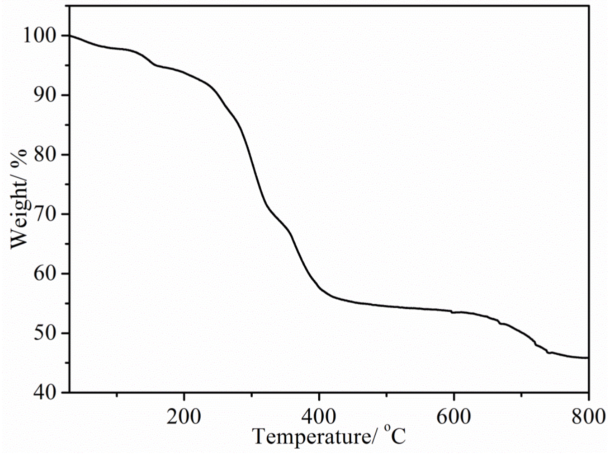

Figure S28. TG curve of compound **TOC-16** in N_2 atmosphere.

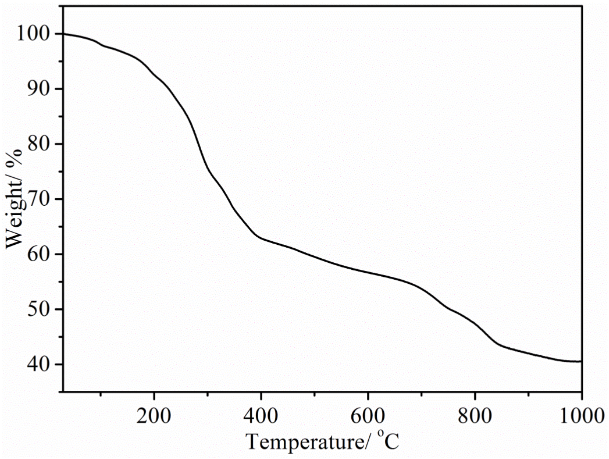

Figure S29. TG curve of compound **TOC-17** in N_2 atmosphere.

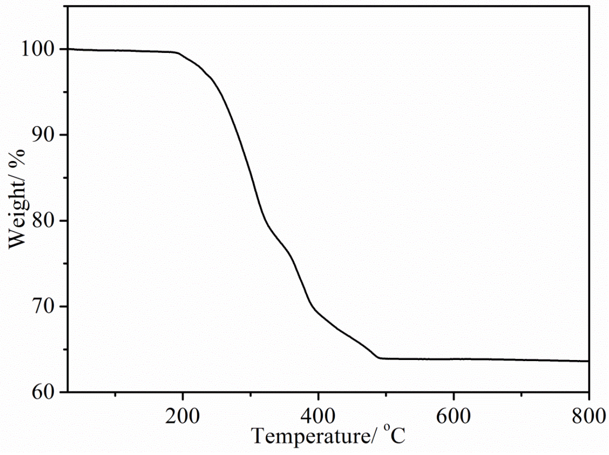

Figure S30. TG curve of compound **TOC-18** in N_2 atmosphere.

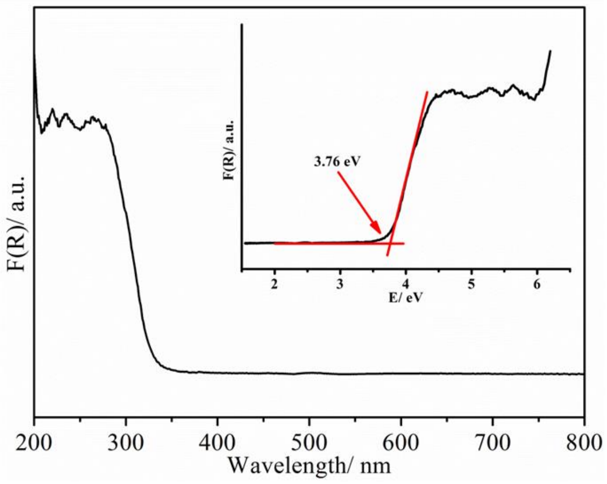

Figure S31. UV-vis diffuse reflectance spectrum of **TOC-12**.

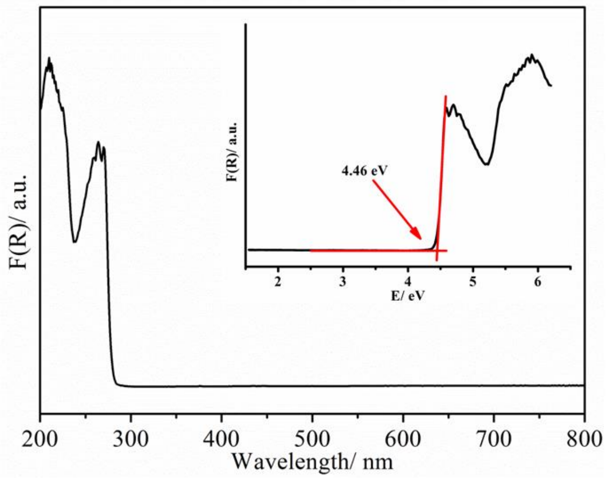

Figure S32. UV-vis diffuse reflectance spectrum of **TOC-13**.

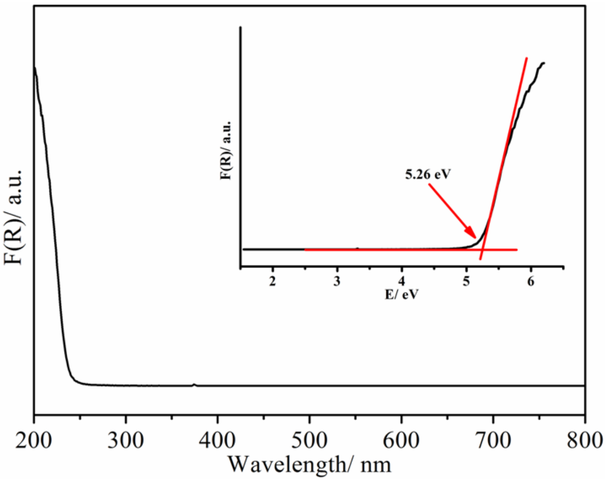

Figure S33. UV-vis diffuse reflectance spectrum of **TOC-14**.

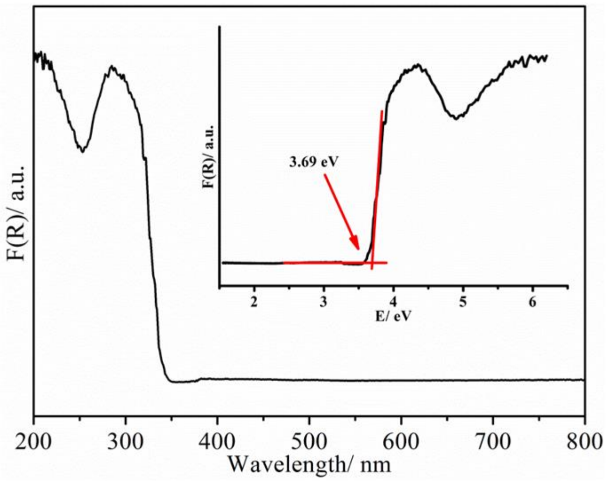

Figure S34. UV-vis diffuse reflectance spectrum of **TOC-17**.

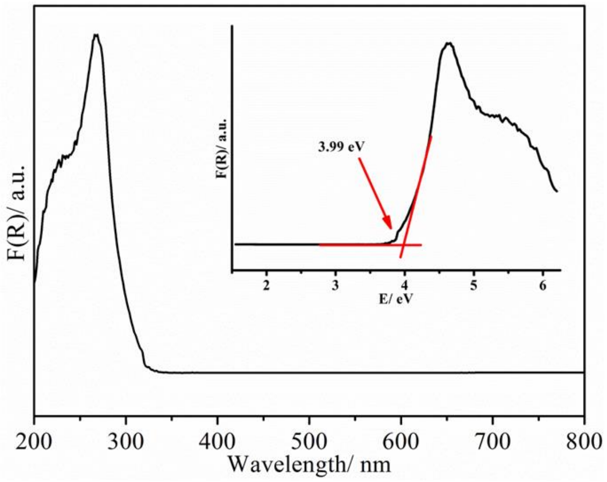

Figure S35. UV-vis diffuse reflectance spectrum of **TOC-18**.

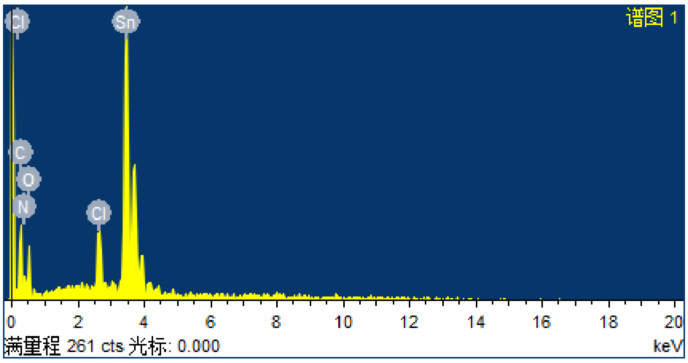

Figure S36. The EDS spectrum of **TOC-15**.

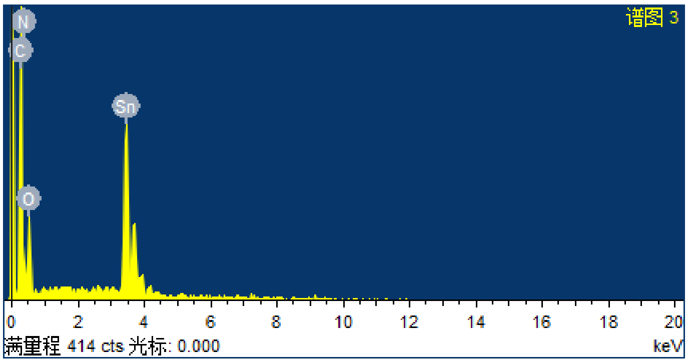

Figure S37. The EDS spectrum of **TOC-16**.

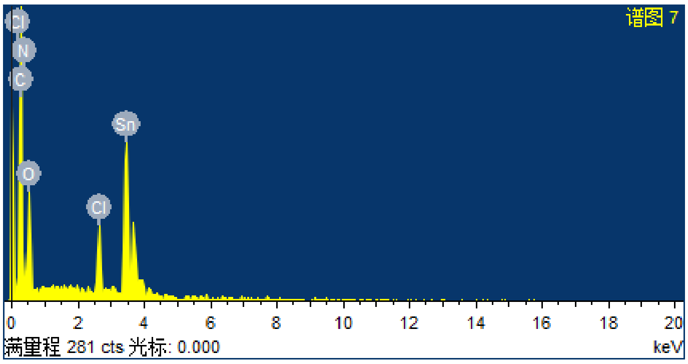

Figure S38. The EDS spectrum of **TOC-17**.

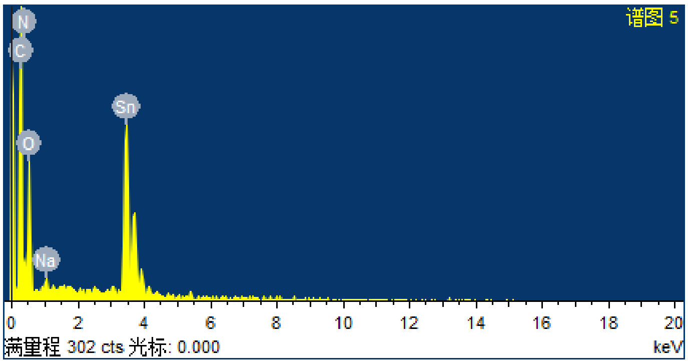

Figure S39. The EDS spectrum of **TOC-18**.

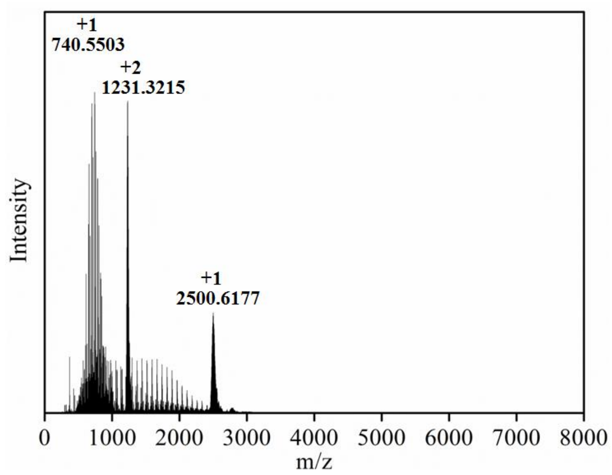

Figure S40. The positive-mode ESI-MS spectrum of **TOC-17** dissolved in ethanol.

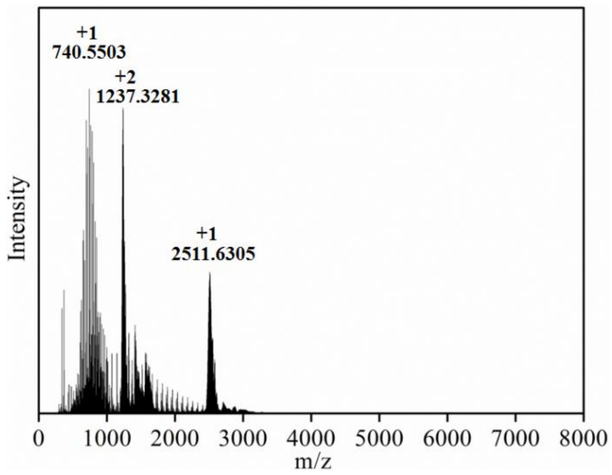

Figure S41. The positive-mode ESI-MS spectrum of mother liquor for **TOC-17** diluted by ethanol.

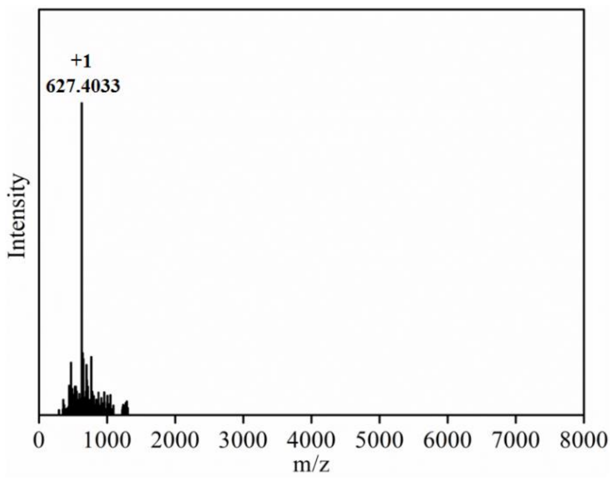

Figure S42. The positive-mode ESI-MS spectrum of **TOC-18** dissolved in ethanol.

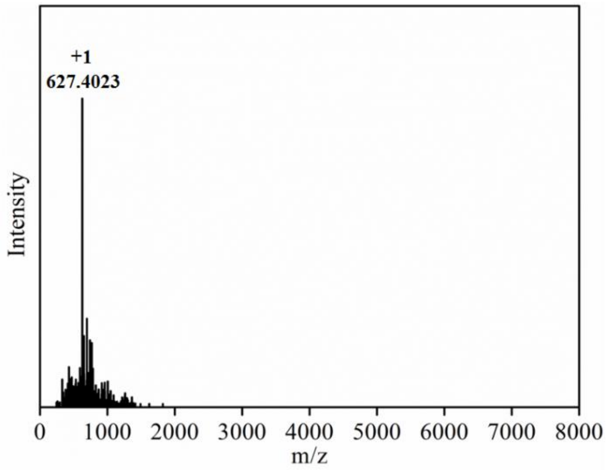

Figure S43. The positive-mode ESI-MS spectrum of mother liquor for **TOC-18** diluted by ethanol.

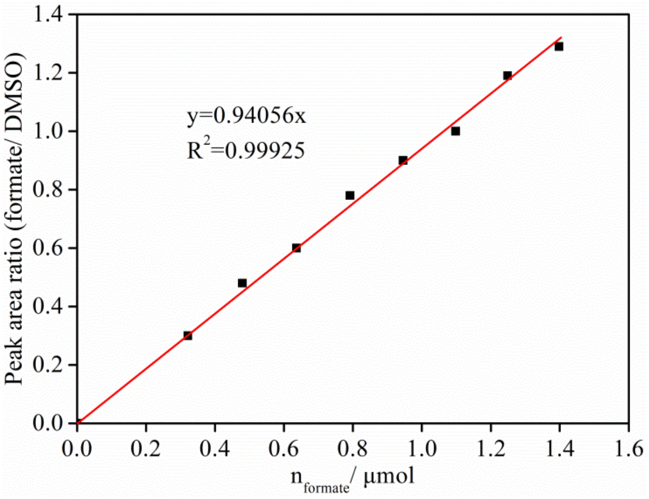

Figure S44. The calibration curve for formate (DMSO as internal standard).

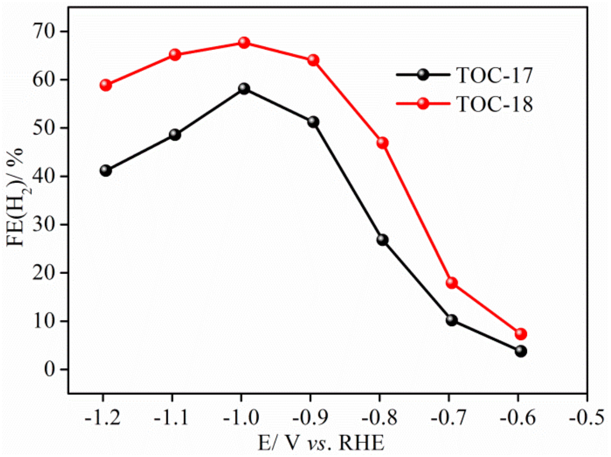

Figure S45. The faradaic efficiencies of H_2 for **TOC-17** and **TOC-18** at various potentials.

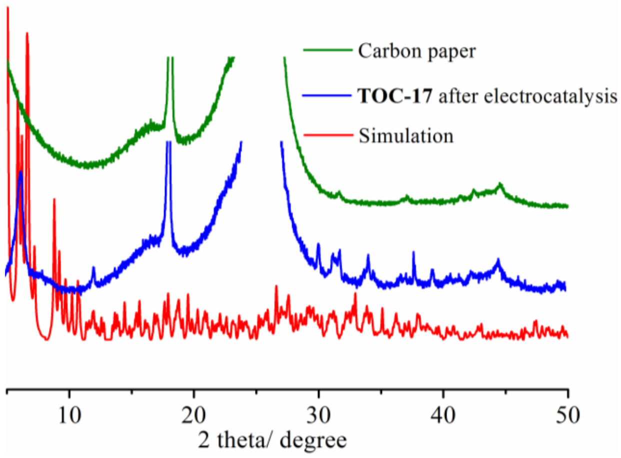

Figure S46. Simulated PXRD pattern of **TOC-17** and experimental PXRD patterns of carbon paper and **TOC-17** on carbon paper after electrocatalysis.

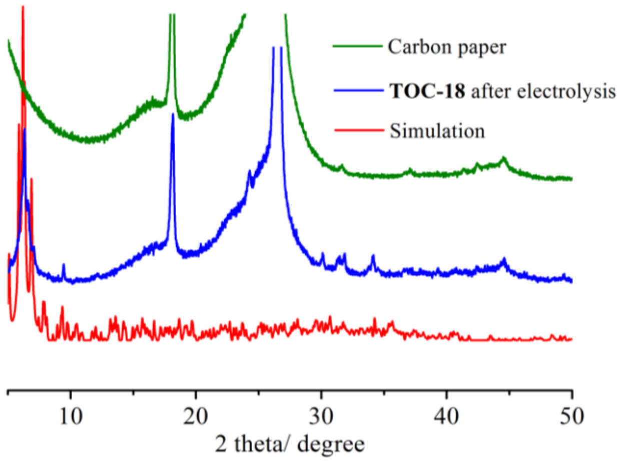

Figure S47. Simulated PXRD pattern of **TOC-18** and experimental PXRD patterns of carbon paper and **TOC-18** on carbon paper after electrocatalysis.
